# Constitutive BAK/MCL1 complexes predict paclitaxel and S63845 sensitivity of ovarian cancer

**DOI:** 10.1038/s41419-021-04073-0

**Published:** 2021-08-12

**Authors:** Dongyan Liu, Xiaonan Hou, Wangyu Wu, Valentina Zanfagnin, Yunjian Li, Cristina Correia, Zhiyang Zhao, Chenggang Zhao, Zhirong Liu, Tao Zhang, Zhiyou Fang, Hongzhi Wang, Chao Xu, Saravut J. Weroha, Scott H. Kaufmann, Haiming Dai

**Affiliations:** 1 Anhui Province Key Laboratory of Medical Physics and Technology, Institute of Health & Medical Technology, Hefei Institutes of Physical Science, Chinese Academy of Sciences, Hefei, 230031 China; 2 University of Science and Technology of China, Hefei, 230026 China; 3 Hefei Cancer Hospital, Chinese Academy of Sciences, Hefei, 230031 China; 4 Division of Medical Oncology, Mayo Clinic, Rochester, MN 55905 USA; 5 Second Affiliated Hospital of Anhui Medical University, Hefei, 230601 China; 6 School of Basic Medical Sciences, Anhui Medical University, Hefei, 230032 China; 7 Department of Molecular Pharmacology and Experimental Therapeutics, Mayo Clinic, Rochester, MN 55905 USA; 8 Division of Oncology Research, Mayo Clinic, Rochester, MN 55905 USA

**Keywords:** Chemotherapy, Apoptosis

## Abstract

We previously found that preformed complexes of BAK with antiapoptotic BCL2 proteins predict BH3 mimetic sensitivities in lymphohematopoietic cells. These complexes have not previously been examined in solid tumors or in the context of conventional anticancer drugs. Here we show the relative amount of BAK found in preformed complexes with MCL1 or BCLX_L_ varies across ovarian cancer cell lines and patient-derived xenografts (PDXs). Cells bearing BAK/MCL1 complexes were more sensitive to paclitaxel and the MCL1 antagonist S63845. Likewise, PDX models with BAK/MCL1 complexes were more likely to respond to paclitaxel. Mechanistically, BIM induced by low paclitaxel concentrations interacted preferentially with MCL1 and displaced MCL1-bound BAK. Further studies indicated that cells with preformed BAK/MCL1 complexes were sensitive to the paclitaxel/S63845 combination, while cells without BAK/MCL1 complexes were not. Our study suggested that the assessment of BAK/MCL1 complexes might be useful for predicting response to paclitaxel alone or in combination with BH3 mimetics.

## Introduction

An increasing number of agents are becoming available for the treatment of ovarian cancer. First-line treatment usually involves surgery (primary or interval debulking) and a paclitaxel/platinum regime, sometimes with bevacizumab. When patients relapse, platinum-sensitive ovarian cancer is treated with more paclitaxel and platinum whereas platinum-resistant disease is treated with bevacizumab plus paclitaxel/doxorubicin/topotecan regime [1]. If these treatments fail, a variety of third-line treatments will be used, including gemcitabine, doxorubicin, or topotecan [2]. More recently, several PARP inhibitors (PARPi), including olaparib, niraparib, and rucaparib, were approved to treat ovarian cancer patients with *BRCA1/2* mutations [1–4], which occur in ~20% of ovarian cancers. About 63% of *BRCA1/2*-mutant ovarian cancers will achieve a complete remission or partial remission after PARPi treatments [5]. Toxicities of these different treatments are substantial, with allergic reactions, myelosuppression, and peripheral neuropathy seen with paclitaxel/carboplatin; hypertension, bleeding and thrombosis with bevacizumab; and myelosuppression as well as possibly the emergence of secondary leukemia with PARPi. Given the variety of treatments available as well as the potential severity of their toxicities, it is very helpful to predict drug sensitivity in ovarian cancer patients before the treatments were applied.

Many methods have been explored to predict the sensitivity of anticancer drugs [6]. Next-generation sequencing techniques and reverse transcription-polymerase chain reaction (PCR), which can obtain information about DNA mutations and RNA expression, are widely used to predict drug sensitivities of many molecularly targeted drugs [7]. As a supplement, analysis of protein levels using immunohistochemistry has also been applied. Additional functional tests [8], including ATP content determination [9], patient-derived organoids [10], organotypic cultures [11, 12], and patient-derived xenografts (PDX) [13], have also been developed to predict anticancer drug sensitivity, although their use is not yet widespread. Moreover, methods used to predict sensitivities of classical chemotherapy drugs are still lacking. Recently, based on (i) the observation that many anticancer drugs induce cancer cell death by activating the mitochondrial apoptosis pathway [14] and (ii) the notion that the primed status of this pathway could reflect the readiness of a cancer cell to undergo drug-induced cell death, the BH3 profiling and the dynamic BH3 profiling (DBP) methods were developed [15].

The mitochondrial apoptosis pathway is controlled by BCL2 family proteins [16]. Among the BCL2 family proteins, two pro-apoptotic members, BAK and BAX can be activated to cause mitochondrial outer membrane permeabilization (MOMP), leading to *cytochrome* c release and subsequent caspase activations. The antiapoptotic family members, including BCLX_L_, MCL1, and BCL2, prevent MOMP by inhibiting activated BAK or BAX. The BH3-only proteins BIM, PUMA, BID, BAD, NOXA, BMF, HRK, and BIK, which act as sensors of various stresses, can induce MOMP through direct activation of BAK or BAX or through the inhibition of antiapoptotic members [16, 17].

Expression of BCL2 family members has been associated with chemotherapy sensitivities of various cancer types. For example, BCL2 over-expression results in paclitaxel resistance in melanoma and breast cancer cells [18, 19]. High BCLX_L_ expression was reported to induce cisplatin and paclitaxel resistance in lung cancer and melanoma [19, 20]. Moreover, low BAX expression associates with poor response of breast cancer to chemotherapy [21].

BH3 profiling and the DBP methods, which are functional analyses using BH3 peptides to induce mitochondrial membrane depolarization, predict leukemia cell sensitivities to several topoisomerase II inhibitors, including etoposide, daunorubicin, and mitoxantrone [15]. Moreover, BH3 profiling has been reported to predict sensitivities of multiple myeloma and several solid tumors to different drugs [22]. While sensitivities to several anticancer drugs can be predicted using this method, new techniques that can simultaneously predict drug responses and provide new mechanistic insight are still needed.

BAK activation involves a series of steps that include (i) a conformational change at the N-terminus of the protein, (ii) externalization of the BAK BH3 domain, (iii) formation of BH3 in-groove homodimers, and (iv) oligomerization of the dimers to form large oligomers that permeabilize the outer mitochondrial membrane [23, 24]. We have previously reported that partially activated BAK, i.e., BAK that has externalized its BH3 domain and become bound to antiapoptotic BCL2 family members, is present in varying amounts in lymphohematopoietic cells [25]. Moreover, the BH3 mimetic sensitivities of these lymphohematopoietic cells reflect the extent and nature of the complexes containing partially activated BAK. In particular, binding of partially activated BAK to BCLX_L_ predicts sensitivity to navitoclax, while binding to MCL1 predicts sensitivity to the MCL1 inhibitor A1210477 [25]. Because BCL2 family proteins play important roles in cell death after many chemotherapies, we hypothesized that the extent and nature of the complexes containing partially activated BAK might predict chemotherapy sensitivity in other cancers as well. In the present study, we found that BAK was partially activated in many ovarian cancer cell lines prior to drug treatment as well. Moreover, the existence of BAK/MCL1 complexes predicted sensitivities of cancer cells to the recently developed MCL1 inhibitor S63845 [26] and the conventional chemotherapy drug paclitaxel. Finally, we also found the S63845 sensitizes a portion of ovarian cancer models to paclitaxel in vitro and in vivo.

## Results

### Partial BAK activation status varies among ovarian cancer cell lines

**Fig. 1:**
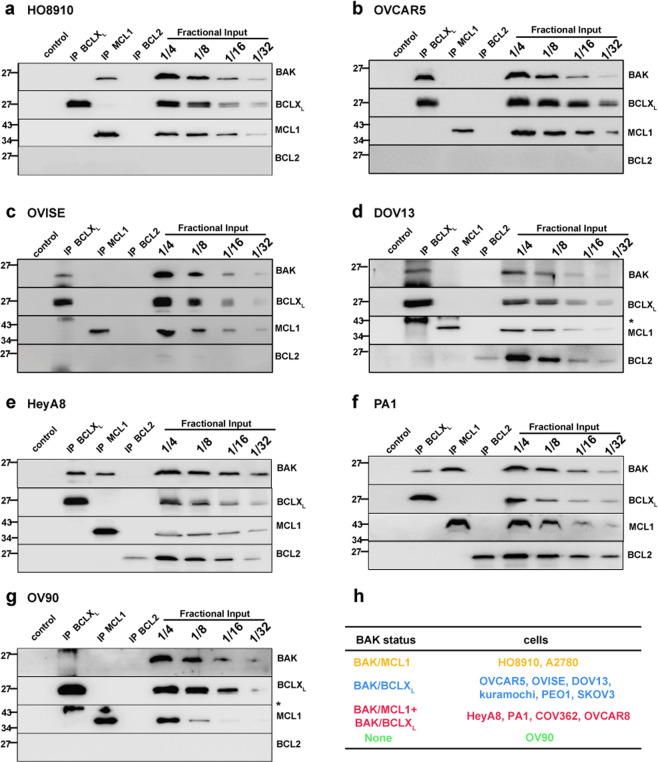
BAK partial activation status varies among ovarian cancer cell lines. **a**–**f** CHAPS lysates of ovarian cancer cell lines HO8910 (**a**), OVCAR5 (**b**), OVISE (**c**), DOV13 (**d**), HeyA8 (**e**), PA1 (**f**), and OV90 (**g**) were immunoprecipitated (IP) with antibodies to BCLX_L_, MCL1, or BCL2, and compared with serial dilutions of the input. IPs with normal rabbit IgG served as negative controls. *, heavy chain. **h** Classification of BAK partial activation status of the ovarian cancer cell lines according to the IP results. BAK status was defined as BAK bound to MCL1 only (BAK/MCL1, in orange), BAK bound to BCLX_L_ only (BAK/BCLX_L_, in blue), BAK bound to MCL1 and BCLX_L_ (BAK/MCL1 + BAK/BCLX_L_, in red), and BAK not bound to any indicated antiapoptotic BCL2 proteins (None, in green), respectively. The boundary-value to define BAK as partially activated was set as 5% of total cellular BAK bound to BCL2, BCLX_L_, or MCL1.

Our previous study suggested that BAK is partially activated in many lymphohematopoietic cell lines, as indicated by its binding to antiapoptotic BCL2 family members using immunoprecipitation assays [25]. To examine whether BAK is also partially activated in ovarian cancer cell lines, BCLX_L_, MCL1, and BCL2 were immunoprecipitated from 13 untreated ovarian cancer cell lines (Fig. 1 and Supplementary Fig. 1), and the percentage of bound BAK was analyzed (Supplementary Fig. 2). This analysis revealed four different patterns. First, in HO8910 and A2780 cells, BAK was predominantly bound to MCL1 (Fig. 1a, h and Supplementary Fig. 1a, g). Second, in several lines, including OVCAR5, OVISE, DOV13, Kuramochi, PEO1, and SKOV3, BAK was predominantly bound to BCLX_L_ (Fig. 1b–d, h and Supplementary Fig. 1b–d, g). Third, in some cell lines, including HeyA8, PA1, COV362, and OVCAR8, BAK was constitutively bound to both BCLX_L_ and MCL1 (Fig. 1e, f, h and Supplementary Fig. 1e–g). Fourth, in the cell line OV90, BAK was not bound to BCLX_L_, MCL1, or BCL2, suggesting BAK is not partially activated (Fig. 1g, h and Supplementary Fig. 1g).

### BAK/MCL1 complexes predict cancer cell sensitivity to paclitaxel and S63845

**Fig. 2:**
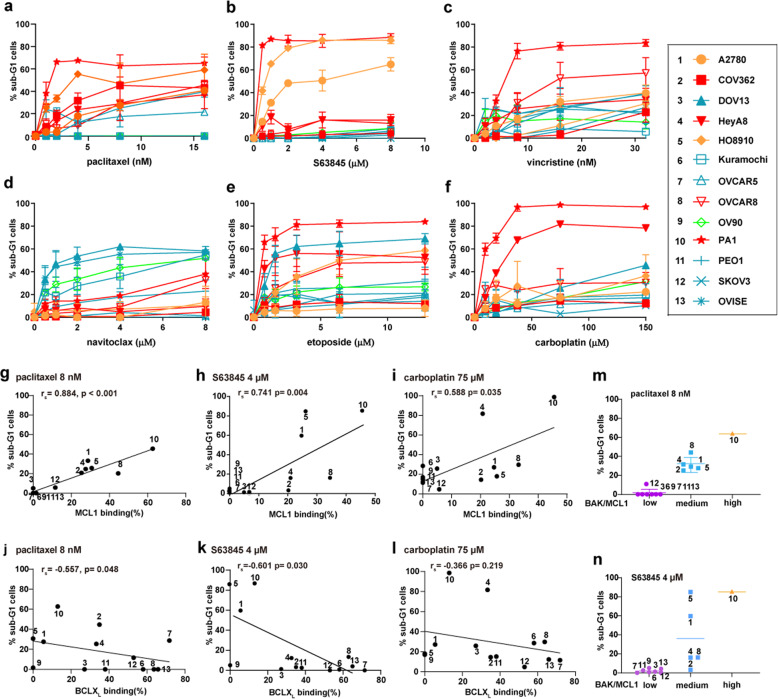
Preformed BAK/MCL1 complexes predict cancer cell sensitivity to paclitaxel and S63845. **a**–**f** After the cell lines were treated with paclitaxel (**a**), S63845 (**b**), vincristine (**c**), navitoclax (**d**), etoposide (**e**), or carboplatin (**f**) for 48 h, the percentages of sub-G1 cells were assessed by flow cytometry (*n* = 3–5 independent experiments, means ± S.D.). Cells with BAK status classified as BAK/MCL1, BAK/BCLX_L_, BAK/MCL1 + BAK/BCLX_L_, and none are indicated with orange, blue, red, and green, respectively. The numbers before each cell line in the Inset will be used to represent the cell lines in Figs. 2,  4,  5, and Supplementary Figs. 4 and 7. **g**–**i** The correlations of BAK/MCL1 complexes and cell death induced by paclitaxel 8 nM (**g**), S63845 4 μM (**h**), and carboplatin 75 μM (**i**) were analyzed using Spearman rank correlation, respectively. **j**–**l** The correlations of BAK/BCLX_L_ complexes and cell death induced by paclitaxel 8 nM (**j**), S63845 4 μM (**k**), and carboplatin 75 μM (**l**) were analyzed using Spearman rank correlation, respectively. **m**, **n** Cell lines were grouped into three different groups according to the percentage of BAK bound to MCL1 (BAK/MCL1), and the apoptosis induced by paclitaxel 8 nM (**m**), or S63845 4 μM (**n**) was indicated for each cell line.

To determine whether BAK partial activation status was associated with sensitivity to anticancer drugs in ovarian cancer cell lines, we assessed the ability of several anticancer drugs to induce apoptosis in the same 13 ovarian cancer cells (Fig. 2a–f and Supplementary Fig. 3a–d). These drugs included paclitaxel [27] and vincristine [28], which alter microtubule dynamics and enhance or inhibit microtubule polymerization, respectively; carboplatin [29], which forms DNA inter- and intra-strand crosslinks; topotecan [30, 31], which selectively poisons topoisomerase I; etoposide [32], which selectively poisons topoisomerase II; 5-FU [33], which is metabolized to an inhibitor of thymidylate synthetase; and olaparib [34], which inhibits multiple poly(ADP-ribose) polymerase (PARP) family members [35]. In addition, we examined sensitivity to multiple BH3 mimetics, including navitoclax, which inhibits BCL2, BCLX_L_, and BCLw [36]; and A1210477 [37] and S63845 [26], which preferentially neutralize MCL1. Many of these drugs have been used for ovarian cancer treatments and the others are under pre-clinical development or clinical trials for ovarian cancer. All of these agents induced dose-dependent apoptosis in ovarian cell lines, as indicated by the sub-G1 populations (Fig. 2a–f and Supplementary Fig. 3a–d) and an increase in active caspase-3/7 (Supplementary Fig. 3e). Moreover, the broad-spectrum caspase inhibitor Q-VD-OPh inhibited the cell death induced by the anticancer drugs (Supplementary Fig. 3f). Sensitivities to these agents varied widely among the ovarian cancer cell lines.

When the relationship between BAK status and anticancer drug sensitivities was examined, the presence of BAK/MCL1 complexes was found to positively correlate with cell deaths induced by paclitaxel (*r*_s_ = 0.884, *p* < 0.001), S63845 (*r*_s_ = 0.741, *p* = 0.004) and carboplatin (*r*_s_ = 0.588, *p* = 0.035) (Fig. 2g–i), while no significant correlation was found between the presence of BAK/MCL1 complexes and cell death induced by vincristine, etoposide, olaparib, topotecan, 5-FU, navitoclax, and A1210477 (Supplementary Fig. 3g). The presence of BAK/MCL1 complexes correlated with cell deaths induced by paclitaxel and S63845 across several different concentrations (Supplementary Fig. 4). On the other hand, the presence of constitutive BAK/BCLX_L_ complexes was only found to significantly negatively correlate with cell death induced by S63845 and paclitaxel, but not to the cell death induced by any other drugs (Fig. 2j–l and Supplementary Fig. 3g).

Because the preformed BAK/MCL1 complexes correlated with cell death induced by both paclitaxel and the MCL1 inhibitor S63845 in ovarian cell lines, we analyzed the correlation between the IC_50_ of paclitaxel and the IC_50_ of MCL1 inhibitor AZD5991 or MIM1 from the Genomics of Drug Sensitivity in Cancer database. As shown in Supplementary Fig. 5, the IC_50_ of paclitaxel showed positive correlations with the IC_50_s of AZD5991 (*r*_p_ = 0.696, *p* < 2.97 × 10^−10^) and MIM1 (*r*_p_ = 0.454, *p* < 2.2 × 10^−16^) across all cell lines, and also across ovarian cancer cell lines only (*r*_s_ = 0.412, *p* = 0.04 for AZD5991, and *r*_s_ = 0.684, *p* = 1.88 × 10^−5^ for MIM1), further supporting our results.

In further analysis, we grouped the cells according to the percentage of total cellular BAK bound to MCL1 [low (<5%), medium (5–40%), and high (>40%)] and analyzed the cell death induced by paclitaxel and S63845 between these groups. As shown in Fig. 2m, n, the cell deaths induced by paclitaxel and S63845 were markedly different among these groups.

### Preformed BAK/MCL complexes associate with paclitaxel sensitivity in ovarian cancer PDX models

**Fig. 3:**
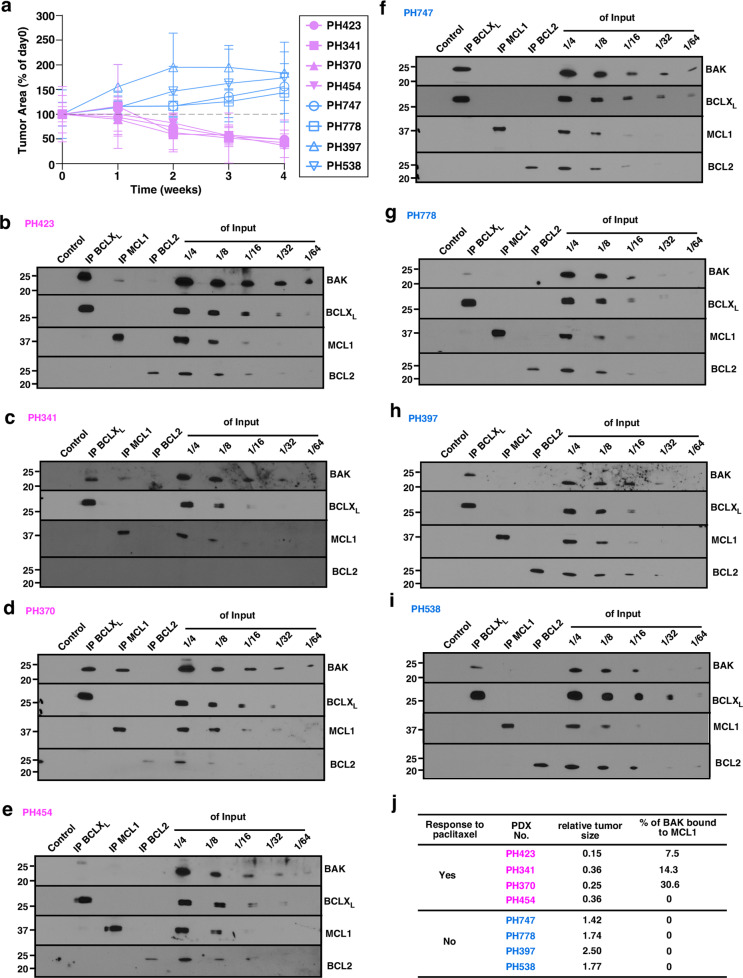
Pre-formed BAK/MCL1 complexes predict paclitaxel sensitivity in PDX models. **a** Four PDX models (PH423, PH341, PH370, and PH454) showed tumor reduction after paclitaxel treatments and four PDX models (PH747, PH778, PH397, and PH538) did not show tumor reduction after paclitaxel treatments. **b**–**i** CHAPS lysates of PDX samples PH423 (**b**), PH341 (**c**), PH370 (**d**), PH454 (**e**), PH747 (**f**), PH778 (**g**), PH397 (**h**), and PH538 (**i**) were immunoprecipitated (IP) with antibodies to BCLX_L_, MCL1, or BCL2, and compared with serial dilutions of the input. Pull-downs with protein G beads only served as a negative control. **j** Relative tumor size as assessed by transabdominal ultrasound after treatment and % BAK bound to MCL1 are summarized for PDX samples in both the paclitaxel responsive group (purple numbers) and paclitaxel unresponsive group (blue numbers). The relative tumor size after treatment was calculated by (tumor area after treatment)/(tumor area before treatment). Relative tumor size ≤0.5 was defined as responsive and relative tumor size >1.0 was defined as unresponsive.

To assess the relationship between partially activated BAK and paclitaxel sensitivity under more physiological conditions, we examined a series of ovarian cancer PDXs. Previous studies have demonstrated that these models, which are grown intraperitoneally, recapitulate many of the features of ovarian cancer from which they are derived, including retention of morphological features, degree of infiltration by stroma, retention of driver mutations, and propensity for inducing ascites and/or bowel obstruction [38, 39]. Eight PDX samples were chosen for these studies, four that were sensitive to paclitaxel and four that were not (Fig. 3a). Among the models that were sensitive to paclitaxel, three out of four samples had detectable BAK/MCL1 complexes prior to any treatment (Fig. 3b–e). In contrast, in the samples that were not sensitive to paclitaxel, 0 out 4 samples had detectable BAK/MCL1 complexes (Fig. 3f–j). Thus, the association between the constitutive presence of BAK/MCL1 complexes and paclitaxel sensitivity also extended to ovarian cancer PDX models.

### BCL2 family expression at the protein and mRNA levels does not predict paclitaxel sensitivity in ovarian cancer

**Fig. 4:**
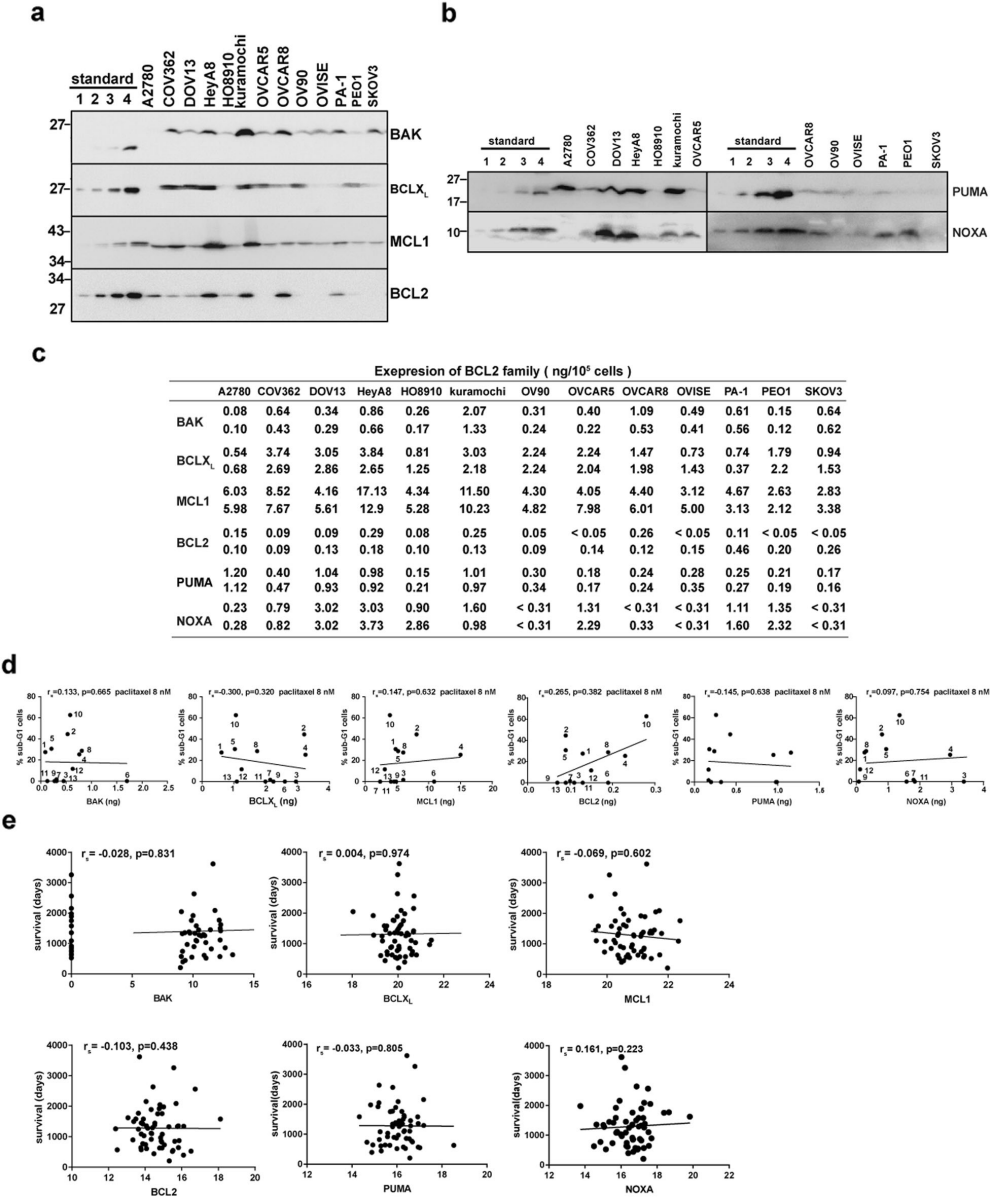
BCL2 family protein or mRNA expression does not predict paclitaxel sensitivity in ovarian cancer. **a**, **b** Whole-cell lysates (1 × 10^5^ cells) of indicated cell lines, along with different concentrations of purified corresponding standard protein, were blotted with antibodies to BAK, BCLX_L_, MCL1, and BCL2 (**a**), or antibodies to PUMA and NOXA (**b**). The amount of standard proteins are: BAK∆TM (0.19, 0.38, 0.75, 1.50 ng); BCLX_L_ ∆TM (1.0, 2.0, 4.0, and 8.0 ng); MCL1∆TM (1.0, 2.0, 4.0, and 8.0 ng); BCL2∆TM (0.05, 0.10, 0.20, and 0.40 ng); PUMA (0.06, 0.13, 0.25, and 0.50 ng); and NOXA (0.31, 0.63, 1.25, and 2.50 ng). **c** BCL2 family proteins evaluated in 10^5^ of indicated ovarian cancer cell lines. Data from two independent experiments were shown. **d** Correlation between protein levels (ng/10^5^ cells) of BAK, BCLX_L_, MCL1, BCL2, PUMA, and NOXA, respectively, and apoptosis induced by 8 nM paclitaxel. **e** Correlation of overall survival with an expression of mRNA encoding BAK, BCLX_L_, MCL1, BCL2, PUMA, and NOXA from TCGA database, respectively. Ovarian cancer patients with paclitaxel treatment but without radiotherapy were selected.

Previous studies have indicated that the expression of BCL2 family proteins can predict anticancer drug sensitivities in breast cancer and melanoma [18, 19, 21]. To test whether BCL2 family protein levels can predict drug sensitivities of ovarian cancer cells, a quantitative western blotting analysis of BCL2 family proteins was performed (Fig. 4a–c). Overall, compared to the acute lymphoid leukemia cell line Jurkat in our previous studies [40], ovarian cancer cells have about 2–8 times more BCLX_L_ and 8–50 times more MCL1, but much less BCL2 (Supplemental Fig. 6a).

When the relationships between BCL2 family protein expression and the induction of apoptosis by anticancer drugs were examined, MCL1 and BCL2 levels were found to correlate with carboplatin-induced cell death, while BCL2 and NOXA levels were found to correlate with etoposide-induced cell death (Supplementary Fig. 6b). However, the protein levels were not found to correlate with cell deaths induced by paclitaxel, S63845, vincristine, navitoclax, olaparib, topotecan, 5-FU, or A1210477 (Fig. 4d and Supplementary Fig. 6b).

To examine whether mRNA levels of BCL2 family proteins could be a predictive marker for ovarian cancer treatment efficacy, ovarian cancer patient data from gene expression profiling interactive analysis (GEPIA) and Kmplot (Kaplan Meier Plot) databases were obtained, and overall survival was analyzed between groups with high expression versus low expression of each BCL2 family member at the mRNA level. This analysis indicated that ovarian cancers with high BCLX_L_ mRNA in GEPIA tended to have shorter survival, and patients with high PUMA mRNA in Kmplot tended to be associated with shorter survival (Supplementary Fig. 6c, d). To further focus on the ovarian cancer patients treated with paclitaxel and to rule out a possible influence by radiotherapy, a total of 59 ovarian cancer patients who received paclitaxel treatment but did not receive radiotherapy from the TCGA database were analyzed. No significant correlation between the mRNA level of the BCL2 family and overall survival was observed in this group of patients (Fig. 4e).

Taken together, these data suggest that the presence of constitutive BAK/MCL1 binding might be a better marker than single BCL2 family protein expression or mRNA expression for the prediction of paclitaxel sensitivity.

### Paclitaxel-induced BIM preferentially binds MCL1 and promotes BAK dissociation in ovarian cell lines

**Fig. 5:**
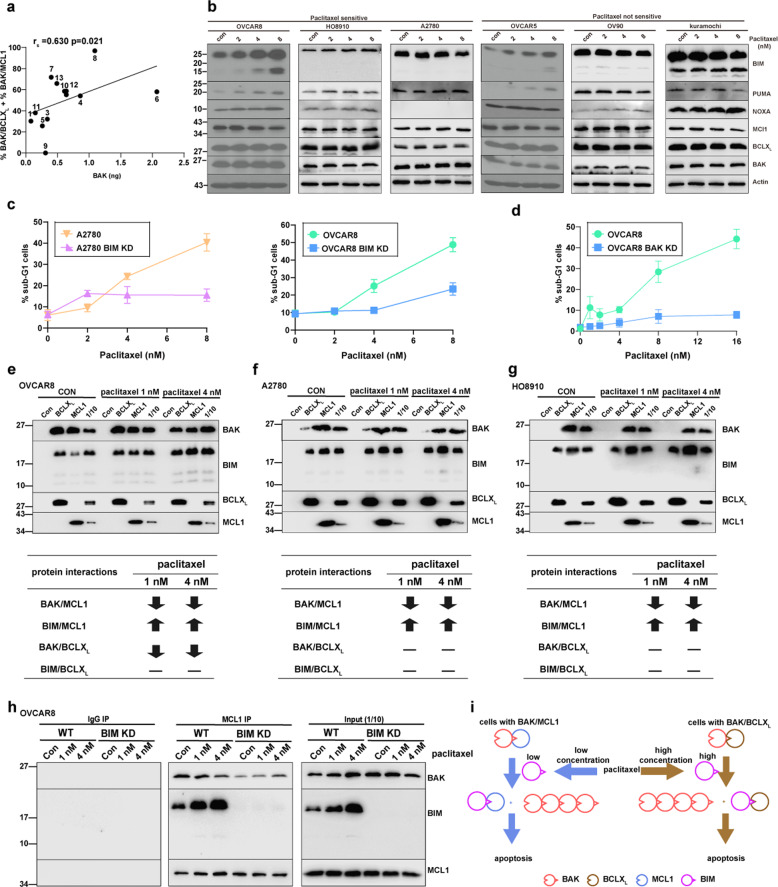
BIM induced by low-dose paclitaxel binds MCL1 and disrupts the BAK/MCL1 complexes. **a** Spearman correlation analysis between protein levels (ng/10^5^ cells) of BAK and %BAK/BCLX_L_ + %BAK/MCL1. **b** After paclitaxel sensitive (OVCAR8, HO8910, A2780) and paclitaxel insensitive cells (OVCAR5, OV90, Kuramochi) were treated with the indicated concentrations of paclitaxel, whole-cell lysates were blotted for indicated proteins. **c**, **d** A2780 (left panel of **c**), or OVCAR8 (right panel of **c**, and **d**) cells were transfected with indicated siRNA to induce protein knockdown (KD). After 24 h, cells were treated with the indicated concentrations of paclitaxel for 48 h, the percentages of sub-G1 cells were detected by flow cytometry. (*n* = 3 independent experiments, mean ± S.D.) **e**–**g** After OVCAR8 (**e**), HO8910 (**f**), or A2780 (**g**) cells were treated with the indicated concentrations of paclitaxel in the presence of Q-VD-OPh for 48 h, CHAPS lysates were immunoprecipitated with rabbit IgG (CON), or antibodies to BCLX_L_ or MCL1. The immunoprecipitated proteins together with the indicated amount of input (1/10 or 1/4) were blotted with the indicated antibodies (upper panels) and analyzed (bottom panels). **h** After OVCAR8 were transfected with BIM siRNA (BIM KD) for 24 h, cells were then treated with the indicated concentrations of paclitaxel in the presence of Q-VD-OPh for 48 h. CHAPS lysates were then prepared for immunoprecipitation with rabbit IgG (CON) or antibodies to MCL1. The immunoprecipitated proteins together with indicated one-tenth of input were blotted with the indicated antibodies. **i** Proposed model to explain why cells with BAK/MCL1 complexes were more sensitive to paclitaxel.

The partial activation of BAK observed in malignant lymphohematopoietic cells appears to be mediated by concentration-dependent BAK auto-activation [25]. In agreement with these earlier results, we found that partial BAK activation in untreated ovarian cancer cell lines is also correlated with BAK protein levels (Fig. 5a), but not with the other BCL2 family proteins (Supplementary Fig. 7a).

Based on current understanding, partially activated BAK will be mainly sequestered by BCLX_L_ or MCL1. Thus, levels of BCLX_L_, MCL1, and the other BH3 only proteins might all have the possibility of influencing whether partially activated BAK will be bound to BCLX_L_ or MCL1. Importantly, none of the BCL2 family proteins including BAK, BCLX_L_, MCL1, BCL2, PUMA, or NOXA significantly correlated with the BAK distribution between BCLX_L_ and MCL1 (Supplementary Fig. 7b), suggesting that multiple factors acting together might influence BAK distribution after externalization of its BH3 domain.

We noticed that the ovarian cancer cells have more abundant BCLX_L_ and MCL1 than the lymphohematopoietic cells we previously characterized (Fig. 4a–c, Supplementary Fig. 6a and ref. [40]). In line with the increased dependence on these antiapoptotic proteins, a combination of navitoclax and S63845 induces cell death in these cell lines, whereas single agent navitoclax or S63845 could not (Supplementary Fig. 8), suggesting BCLX_L_ and MCL1 both need to be neutralized before apoptosis will occur.

Previous studies have indicated that the BH3-only protein BIM is the major mediator of paclitaxel-induced apoptosis in epithelial tumor cells [41]. To study the BCL2 family proteins involved in paclitaxel-induced apoptosis in ovarian cancer cells, we treated six ovarian cancer cell lines, three that are sensitive and three that are insensitive to paclitaxel-induced killing (Supplementary Fig. 9a), and assessed changes of BCL2 family proteins by western blotting (Fig. 5b). We observed paclitaxel-induced BIM upregulation in two of three paclitaxel-sensitive cell lines and one of three paclitaxel insensitive lines (Supplementary Fig. 9b), suggesting that BIM upregulation was not the only factor accounting for paclitaxel sensitivity in these cell lines. We also observed PUMA upregulation in OVCAR8, HO8910, and OVCAR5 cells, while no obvious change was observed for other BCL2 family proteins (Fig. 5b). BIM knockdown inhibited paclitaxel-induced apoptosis in both OVCAR8 and A2780 (with or without BIM upregulation after paclitaxel treatment), reflecting the important role of BIM in paclitaxel-induced apoptosis (Fig. 5c). BAK knockdown also inhibited paclitaxel-induced apoptosis in OVCAR8 cells (Fig. 5d).

Because BIM can also be released from dynein light chain 2 to induce apoptosis upon paclitaxel treatment [42], further experiments examined whether the BCLX_L_ or MCL1 bound BIM was increased. In all the three paclitaxel sensitive cell lines (OVCAR8, HO8910, and A2780), we observed increased BIM bound to MCL1, but not to BCLX_L_, after paclitaxel treatment, suggesting that BIM mobilized by paclitaxel (either through upregulation or dissociation from dynein light chain [42]) binds to MCL1 in preference to BCLX_L_. The binding of BIM also displaces BAK from MCL1 (Fig. 5e–g, and Supplementary Fig. 9c–e). Moreover, in the BIM knockdown cells, BAK was not released from MCL1 upon paclitaxel treatment (Fig. 5h). Taken together, these data suggest a model in which paclitaxel induces BIM expression or frees BIM from dynein light chain 2, and BIM displaces BAK to induce killing (Fig. 5i).

### S63845 synergizes with paclitaxel in ovarian cancer cells with preformed BAK/MCL1 complexes in vitro

High doses of paclitaxel have been reported to induce severe side effects, including marrow suppression, peripheral neuropathy, and cardiac rhythm disturbances [43, 44]. Accordingly, sensitizing cancer cells to paclitaxel would potentially be helpful to limit the side effects in the clinic. The data in Fig. 2m, n indicates that cells with high levels of preformed BAK/MCL1 complexes are very sensitive to paclitaxel and S63845 and cells bearing medium BAK/MCL1 complexes are somewhat sensitive to paclitaxel and S63845.

**Fig. 6:**
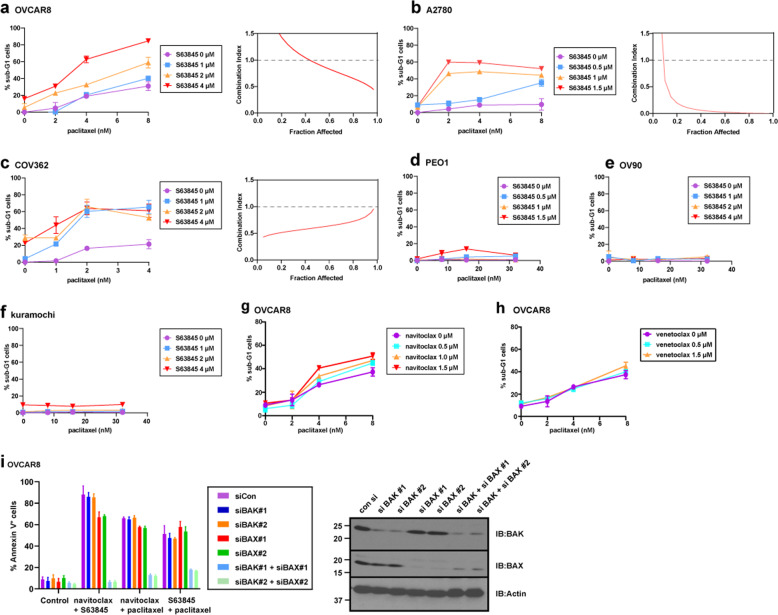
S63845 and paclitaxel synergize in cells with BAK/MCL1 complexes. **a**–**f** After OVCAR8 (**a**), A2780 (**b**), COV362 (**c**), PEO1 (**d**), OV90 (**e**), and Kuramochi (**f**) were treated with paclitaxel in combination with S63845 at the indicated concentrations for 48 h, the percentages of sub-G1 cells were detected by flow cytometry. Right panels in **a**–**c**, combination indices (CIs) were calculated according to the method of Chou and Talalay [60] using all apoptosis data obtained from the left panels. This method calculates the CI, a parameter that indicates whether drug concentrations needed to produce a particular level of cell killing (% affected), are lower than, equal to, or greater than concentrations predicted to have additive effects. Thus, CI < 1 indicates synergy, CI = 1 indicates additivity, and CI > 1 indicates antagonism. **g**, **h** After OVCAR8 cells were treated with paclitaxel combined with navitoclax (**g**) or paclitaxel combined with venetoclax (**h**) at the indicated concentrations for 48 h, the percentages of sub-G1 cells were detected by flow cytometry. **i** Twenty-four hours after OVCAR8 cells were transfected with indicated siRNAs, cells were treated with navitoclax + S63845, navitoclax + paclitaxel, or S63845 + paclitaxel for 48 h, and assayed for Annexin V binding by flow cytometry. (*n* = 3, independent experiments, mean ± S.D.).

Because the amount of BAK/MCL1 complexes plays a role in paclitaxel sensitivity, we hypothesized that the MCL1 inhibitor S63845 might promote paclitaxel-induced cell death. Cell lines that are somewhat sensitive to single-agent paclitaxel and have medium levels of preformed BAK/MCL1 complexes (OVCAR8, A2780, and COV362) or not sensitive and do not have BAK/MCL1 complexes (PEO1, OV90, and Kuramochi) were evaluated. We found that S63845 synergized with paclitaxel to induce apoptosis in OVCAR8, A2780, and COV362 cells, which have preexisting BAK/MCL1 complexes (Fig. 6a–c). In contrast, S63845 failed to enhance paclitaxel-induced apoptosis in cells that do not have BAK/MCL1 complexes (Fig. 6d–f). We also assessed the ability of navitoclax and S63845 to sensitize OVCAR8 cells to vincristine and observed similar results (Supplementary Fig. 10). Thus, whether S63845 can sensitize the cells to paclitaxel correlates with the existence of preformed BAK/MCL1 complexes.

Further experiments indicated navitoclax but not venetoclax could also sensitize OVCAR8 cells to paclitaxel-induced apoptosis (Fig. 6g, h), in agreement with the results that most ovarian cancer cells have abundant BCLX_L_, but not BCL2 (Fig. 4c). Moreover, only BAK and BAX double knockdown, but not the single knockdowns, could inhibit apoptosis induced by paclitaxel combined with navitoclax or S63845 (Fig. 6i and Supplementary Fig. 11), suggesting the cell death induced by the combination can be mediated through either BAK or BAX activation in these cells.

### S63845 synergizes with paclitaxel to inhibit ovarian cancer cells with BAK/MCL1 complexes in vivo

**Fig. 7:**
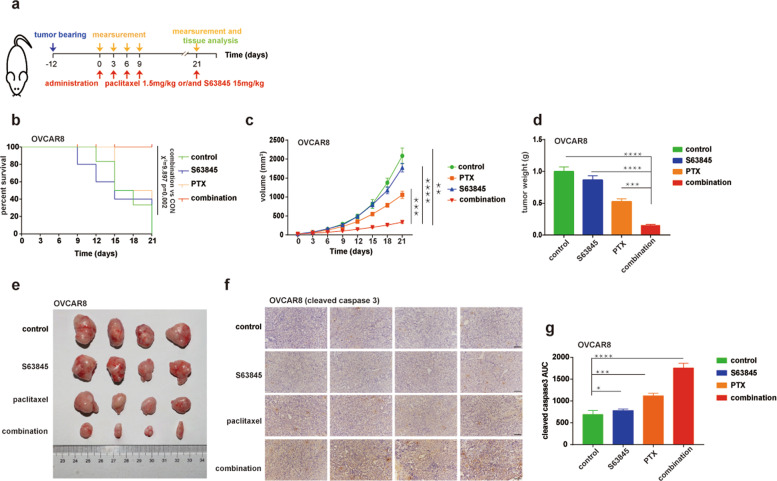
The paclitaxel/S63845 combination inhibits the growth of xenografts bearing BAK/MCL1 complexes. **a** Diagram of this assay. **b** Kaplan–Meier survival curve of OVCAR8 inoculated mice. Each group was treated with or without paclitaxel (1.5 mg/kg) and/or S63845 (15 mg/kg) as indicated in panel (**a**). COX proportional hazards model for paclitaxel + S63845 vs. control was *χ*^2^ = 9.897 (*p* = 0.002), while no significance was observed for paclitaxel or S63845 vs. control (*n* = 10). **c**, **d** Tumor volume (**c**) and weight (**d**) of each group (*n* ≥ 4 mice in each group at the start of treatment, mean ± S.E.M.) observed in OVCAR8 tumor-bearing mice after the start of treatment with vehicle (control), S63845, paclitaxel, and paclitaxel + S63845. **e** Tumors from the control group, S63845 group, paclitaxel group, and combination group were obtained for comparison from OVCAR8 tumor-bearing mice at day 21. **f**, **g** Cleaved-caspase 3 by IHC using tumor tissue from OVCAR8 tumor-bearing mice at day 21 (**f**) and the intensity profiling of DAB stained image were calculated using ImageJ program (**g**). All images are captured at 20× magnification. (*n* = 4, mean ± S.D.) Scale bar in panel **f** 200 μm. Statistical significance in panels **c**, **d**, and **g** was determined by a two-sided unpaired Student’s *t* test. *p < 0.05; **p < 0.01; ***p < 0.001; ****p < 0.0001.

The paclitaxel/S63845 combination was further tested for synergy in vivo (Fig. 7a). In mice inoculated with OVCAR8, the combination displayed significant anticancer effects, while no significant improvement of the overall survival was observed when the mice were treated with either single drug (Fig. 7b). The tumor volume, tumor weight and tumor sizes were also significantly inhibited in the combination group, compared to control, paclitaxel only, or S63845 only groups (Fig. 7c–e). Further, the percentage of OVCAR8 cells that stained positive for cleaved caspase 3 increased significantly in xenografts treated with paclitaxel in combination with S63845 (Fig. 7f, g), suggesting cells were dying through the apoptosis. Taken together, these data suggest that the paclitaxel/S63845 combination also inhibits ovarian cancers with BAK/MCL1 complexes in vivo at achievable drug concentrations.

## Discussion

Since its introduction 25 years ago, paclitaxel has become widely used for the treatment of breast and ovarian cancer. Indeed, it is almost universally administered to ovarian cancer patients as part of a carboplatin/paclitaxel combination [45]. In addition, it retains some activity when ovarian cancer becomes platinum-resistant [46]. Accordingly, there is substantial interest in understanding the antineoplastic action of paclitaxel and being able to predict paclitaxel sensitivity. Here, we have shown that the presence of preformed BAK/MCL1 complexes is a predictor of paclitaxel sensitivity in ovarian cancer cell lines and PDX.

Earlier studies showed that paclitaxel binds to β-tubulin and disrupts microtubule dynamics, leading to mitotic arrest followed by mitotic exit and apoptosis [47, 48]. Additional studies implicated the BH3-only protein BIM in paclitaxel-induced death [49] and suggested that it is either synthesized in response to paclitaxel [50] or released from dynein light chain 2 on microtubules [42]. Diminished BIM expression, but not mitotic slippage, plays an important role in mediating paclitaxel resistance [51]. However, total cellular BIM levels have not proven useful for predicting paclitaxel sensitivity [52]. Consistent with these results, we did not find a significant correlation between BCL2 family protein expression and paclitaxel-induced apoptosis in ovarian cancer cell lines (Fig. 4d). Likewise, BCL2 family expression at the mRNA level also failed to correlate with overall survival in ovarian cancer patients treated with paclitaxel in the TCGA database (Fig. 4e).

Previous studies in hematological malignancies have suggested that cytotoxicity to anticancer agents is related to pre-existing complexes of pro- and antiapoptotic proteins [25, 53, 54]. In this study, we found that the presence of preformed BAK/MCL1 complexes in ovarian cancer significantly correlates with S63845 and paclitaxel sensitivities. Moreover, we found that sensitivity to paclitaxel also correlates with sensitivity to the MCL1 inhibitors MIM1 and AZD5001 (Supplementary Fig. 4). Curiously, we did not find that the preformed BAK/MCL1 complex correlates with another MCL1 inhibitor A1210477. This could reflect the lower affinity of A1210477 for MCL1, confounding effects of some factors such as differential cellular uptake, or an unidentified off-target effect of A1210477. Importantly, the relationship between BAK/MCL1 complexes and response to paclitaxel was not only observed in tissue culture cell lines, but also in PDX models. Notably, the presence of these complexes could not be discerned by measuring BAK expression at the mRNA or protein level but required the analysis of BAK interacting proteins. If this result is confirmed in additional studies, it is possible that the constitutive presence of BAK/MCL1 complexes could be evaluated as a possible pre-selection criterion for ovarian cancers more likely to respond to paclitaxel.

Further studies examined why cells bearing BAK/MCL1 complexes are more sensitive to paclitaxel. We observed similar BIM induction in sensitive ovarian cancer lines and in some cell lines that are not sensitive (Fig. 5b), ruling out differential BIM induction as the sole explanation for differences in paclitaxel sensitivity. Instead, immunoprecipitation assays indicated that the BIM, whether induced by upregulation or released from dynein light chain 2 by low paclitaxel concentrations, binds to MCL1 in preference to BCLX_L_, leading to the displacement of partially activated BAK from MCL1 so that it can be fully activated (Fig. 5e–g, and Supplementary Fig. 9c–e). BIM has also previously been found to preferentially interact with MCL1 than BCLX_L_ or BCL2 under some other conditions [55]. Thus, these observations provide a possible explanation for the heightened paclitaxel sensitivity of cells bearing preformed BAK/MCL1 complexes (Fig. 5i).

Interestingly, we have observed that BAK knockdown markedly inhibited paclitaxel-induced apoptosis in OVCAR8 cells (Fig. 5d), whereas inhibition of paclitaxel/S63845-induced apoptosis requires knockdown of both BAX and BAK (Fig. 6i). In assessing this apparent paradox, it is important to realize that most ovarian cells have an abundance of both BCLX_L_ and MCL1, with particularly high MCL1 levels in OVCAR8 cells (Fig. 4c). Moreover, published results suggest that BIM has a higher affinity for MCL1 than for BAK [56, 57], explaining the tendency of BIM to displace partially activated BAK from MCL1 rather than activate more BAK. While small amounts of BAK and BAX might be activated directly by paclitaxel-mobilized BIM, this direct activation seemingly plays a small role, as indicated by the inability of BAX to support apoptosis induced by paclitaxel alone (Fig. 5d). In contrast, when cells are treated with S63845 in addition to paclitaxel, BIM will no longer be neutralized by MCL1. Once binding sites on BCLX_L_ are exhausted, BIM would potentially be able to activate BAX and BAK, which will contribute more to killing by the combination. Accordingly, it is not surprising that BAX and BAK both need to be knocked down to impact killing by the combination.

Several previous studies have suggested that MCL1 over-expression will induce navitoclax resistance but favor S63845 sensitivity [26, 36]. However, we did not observe a significant correlation between the expression of BCL2 family members and navitoclax- or S63845-induced apoptosis (Supplementary Fig. 6b). Instead, we observed that the existence of preformed BAK/MCL1 complexes also correlates with S63845-induced apoptosis in ovarian cancer cells (Fig. 2h and Supplementary Fig. 3g), in agreement with previous studies in hematopoietic cells [25], suggesting there is a difference between the existence of BAK/MCL1 complexes and merely having high MCL1 expression. Our additional analysis did not find any single BCL2 family protein that determined whether partially activated BAK bound to BCLX_L_ or MCL1 (Supplementary Fig. 7b), which suggests that multiple factors might work together to determine the binding partner for partially activated BAK. This multifactorial determination of the BAK binding partner seemingly explains why the expression of single BCL2 family members at the mRNA or protein level did not predict sensitivities to paclitaxel, S63845, or navitoclax.

Because high paclitaxel doses can cause profound neurological side effects, the observed synergy with S63845 is potentially interesting; it might provide a strategy for diminishing paclitaxel doses but maintaining the antitumor effects. Our results suggest that the pre-formed BAK/MCL1 complexes not only correlate with sensitivity to paclitaxel and S63845 in ovarian cancer cells but also predict synergy of the paclitaxel/S63845 combination (Fig. 6). The predictive value of these complexes reflects the fact that S63845 cannot displace BAK from MCL1 to enhance paclitaxel-induced apoptosis if cells do not have BAK/MCL1 complexes.

Although our results have demonstrated an important role of pre-formed BAK/MCL1 complexes in sensitizing ovarian cancer cells to paclitaxel, S63845, and the combination, there are also some limitations of our study. First, we have only tested eight PDX models, four sensitive to paclitaxel and four not sensitive. Further large-scale testing is needed before BAK/MCL1 complexes can be more widely used to predict drug sensitivities. Second, because the immunoprecipitation assay used in this study will limit the widespread testing of BAK/MCL1 complexes, better methods need to be developed to detect BAK/MCL1 complexes, especially at the level of tumor tissue.

In summary, we have found that pre-existing BAK/MCL1 complexes contribute to apoptotic sensitivity of ovarian cancer to paclitaxel, S63845 and the combination. These studies also establish the importance of MCL1, as well as BAK, as determinants of drug sensitivity in ovarian cancer and provide a potential rationale for further preclinical and possible clinical testing of MCL1 antagonists in ovarian cancer. Moreover, they provide the impetus for further examination of preformed complexes between BAK and MCL1 as potential determinants of anticancer drug sensitivity.

## Materials and methods

### Materials

Reagents were obtained as follows: Navitoclax and A1210477 from MedChemExpress; venetoclax and S63845 from Chemietek; topotecan from Toronto Research Chemicals; olaparib, etoposide, and vincristine from Selleck Chemicals; 5-fluorouracil from Tokyo Chemical Industry; and CHAPS from Millipore-Sigma. Antibodies were purchased from the following suppliers: anti-PUMA (#sc-374223, 1:1000) and anti-actin (goat polyclonal, I-19, #sc-1615, 1:500) antibodies from Santa Cruz Biotechnology; anti-NOXA antibody from ENZO Life Sciences (#ALX-804-408-c100, 1:1000); anti-BCL2 antibody from DAKO (#M0887, 1:1000); anti-BAK antibody from Millipore (#06-536, 1:1000) and antibodies to BAX (#2772S, 1:1000), BCLX_L_ (#2764S, 1:1000), BIM (#2933S, 1:1000), MCL1 (#4572S, 1:1000), caspase-3 (#9662, 1:1000), caspase-9 (#9502, 1:1000), and tubulin (#2148, 1:1000) from Cell Signaling Technology. Rat anti-BID antibody was a kind gift from David Huang (Walter & Eliza Hall Institute, Melbourne, Australia).

### Protein expression and purification

Plasmids encoding BCL2ΔTM, BCLX_L_ΔTM, PUMA, NOXA, MCL1ΔTM, or BAKΔTM were cloned in pET29b (+) [40, 58]. After *Escherichia coli* strain BL21 bearing protein-expressing plasmids were grown to OD600 0.8 and induced by 1 mM isopropyl 1-β-d-thiogalactopyranoside for 24 h at 16 °C (BAKΔTM, BCL2ΔTM, BCLX_L_ΔTM, or MCL1ΔTM) or 5 h at 37 °C (PUMA, NOXA), bacteria were washed, and sonicated on ice in TS buffer (150 mM NaCl containing 10 mM Tris-HCl at pH 7.4, 1 mM PMSF). Proteins were then purified on a Ni^2^^+^-NTA column.

### Cell culture

Ovarian cancer cell lines HeyA8, DOV13, and PEO1 were grown in DMEM containing 100 U/mL penicillin G, 100 μg/mL streptomycin, 2 mM glutamine, and 10% fetal bovine serum (FBS). A2780, COV362, HO8910, Kuramochi, OVCAR5, OVCAR8, OVISE, PA1, and SKOV3 were grown in RPMI 1640 containing 100 U/mL penicillin G, 100 μg/mL streptomycin, 2 mM glutamine, and 10% FBS. All cell lines have been recently authenticated by STR profiling and tested for mycoplasma contamination.

### Immunoprecipitation

For immunoprecipitation assays after treatment, three independent experiments were done. All immunoprecipitations were performed after a 48-h exposure to a drug or diluent in the presence of 5 µM Q-VD-OPh to inhibit caspase activities. Antibody to BCL2, MCL1, or BCLX_L_ was crosslinked to protein G-agarose as previously described [58]. After harvest, cells were lysed at 4 °C in CHAPS lysis buffer (20 mM HEPES, 150 mM NaCl, 1% (w/v) CHAPS and 1% (v/v) glycerol, 1 mM PMSF, 10 μg/mL leupeptin, 10 μg/mL pepstatin, 100 mM NaF, 10 mM sodium pyrophosphate, 1 mM sodium vanadate, and 20 nM microcystin, pH 7.4) for 30 min. After centrifugation at 14,000*g* for 15 min, lysates (containing 500 μg of protein) were incubated for 24 h with pretreated protein G-agarose. Beads were then washed with 1% CHAPS for three times and eluted with SDS sample buffer for immunoblotting. Serial dilutions of whole-cell lysates were used to calculate the percentage of BAK bound to BCL2, BCLX_L_, or MCL1 [25].

### Apoptosis assay

All cells were seeded 24 h before treatment. At a confluence of about 10–15%, cells were treated with indicated drugs or diluents for 48 h, harvested and washed twice with PBS, permeabilized with 0.1% Triton X-100 and stained with propidium iodide. After 20,000 events were collected on a Beckman CytoFLEX flow cytometer, the percentage of sub-G1 events was quantitated using Beckman software. To compare apoptosis induced in different cell lines, 3–5 independent experiments were performed. The diluent-induced cell death was subtracted from each sample and drug-induced cell death was calculated using the formula: (death_observed_ − death_control_)/(1 − death_control_) × 100%.

Alternatively, in combination assays, cells were harvested, washed with PBS, and stained with APC-labeled Annexin-V. Cells were then analyzed on a Beckman CytoFLEX flow cytometer. Three independent experiments were performed.

### Caspase-3/7 activity

All cells were seeded 24 h before treatment. At a confluence of about 10–15%, cells were treated with indicated drugs or diluents for 48 h, harvested and washed twice with PBS, stained with active caspase-3/7 probe (DEVD peptide conjugated to a nucleic acid binding dye, Invitrogen). After 20,000 events were collected on a Beckman CytoFLEX flow cytometer, the percentage of active caspase-3/7+ cells was quantitated using Beckman software. Three independent experiments were performed.

### Immunoblotting

Samples were separated on sodium dodecyl sulfate-polyacrylamide gel electrophoresis gels, transferred to a nitrocellulose membrane, probed with the indicated primary antibodies, and detected with peroxidase-coupled secondary antibodies. Chemiluminescence signals were visualized using a Tanon 3500 gel imaging system and quantified by ImageJ software. Serial dilutions of purified proteins were used as standards to determine the protein levels in the cell lines. After the bands on the western blots were acquired by ImageJ, a linear equation was established from the standard proteins. Relative protein levels were then calculated according to this standard curve.

### siRNA transfections

To knock down BAK or BAX, two sets of siRNAs were used. The sequences of BAK siRNAs were 5′-GTACGAAGATTCTTCAAAT-3′ and 5′-CCCATTCACTACAGGTGAA-3′; the sequences for BAX siRNAs were 5′-GACGAACTGGACAGTAACA-3′ and 5′-TATGGAGCTGCAGAGGATG-3′, respectively [59]. The sequence of BIM siRNA was 5′-GACCGAGAAGGTAGACAAT-3′. Twenty-four-hour after transfection, cells were treated as indicated and then assayed by flow cytometry.

### Bioinformatics analysis

Patient information was obtained from TCGA, GEPIA (http://gepia2.cancer-pku.cn/#survival), and Kaplan Meier Plot (https://kmplot.com/analysis/). Correlation analysis between mRNA levels of BCL2 family proteins and patient survival were then analyzed from Kaplan Meier Plot and GEPIA. Alternatively, patients (treated with paclitaxel and without radiation therapy) from TCGA (TCGA-OV.htseq_fpkm-uq.tsv., TCGA-OV.GDC_phenotype.tsv and TCGA-OV.survival.tsv) were analyzed by Pearson rank correlation.

### Animal study

The animal studies of combination treatments were approved by the Ethics Committee of the Hefei Institutes of Physical Science, Chinese Academy of Sciences. BALB/c-nude mice were originally purchased from Charles River. Five- to six-week-old male mice were housed with a 12 h light–dark cycle (light on 6 a.m.–6 p.m.) with free access to food and water. To establish xenografts, mice were inoculated with 0.1 ml of a 1:1 mixture containing 10^7^ OVCAR8 cells in growth medium and matrigel (BD Biosciences). After 12 days, when the volume of OVCAR8 tumors was about 100 mm^3^, mice were randomly (no particular randomization method was used) divided into four groups (*n* = 10 for each group): Control, paclitaxel treated (PTX, 1.5 mg/kg IV), S63845 treated (IV, 15 mg/kg), and PTX (1.5 mg/kg) + S63845 (15 mg/kg) treated. Both PTX and S63845 were administered every three days for 21 days. Tumor and weight were blindly measured (measured by another person who did not know the groups) every three days. All animals were included in the measurements.

The formula (tumor volume = *ab*^2^/2, where *a* is the length, and *b* is the width) was used to evaluate tumor volume. The trial was terminated when 40% of control mice reached the criteria for euthanasia.

### PDX models

All studies with human samples were approved by Mayo Clinic Institutional Review Board (IRB). All animal studies were carried out in accordance with the relevant guideline and regulations of the Mayo Clinic Institutional Animal Care and Use Committee. Fresh tissues from patients with ovarian or fallopian tube cancer were collected at the time of primary debulking surgery at Mayo Clinic, Rochester. Written informed consent was obtained from all patients and documented in the electronic medical record. All tissues were coded with a patient heterotransplant (PH) number to protect patient identity in accordance with the Mayo Clinic IRB and Health Insurance Portability and Accountability Act regulations. PDXs were developed by intraperitoneal injection of the donor tumor into female SCID beige mice (C.B-17/IcrHsd-PrkdcscidLystbg-J; Envigo, Indianapolis, IN).

For all PDX studies, cryogenically preserved human ovarian cancer tumors were rapidly thawed and reestablished in female SCID Beige mice as previously described [38]. Briefly, 0.1–0.2 cc of the minced tumor was prepared in 1:1 ratio with McCoy’s 5A Modified Medium McCoy’s media before intraperitoneal injection. Low-passage models (≤5 to minimize genetic drift) were used for pacitaxel experiments. Tumor cross-sectional areas were followed by transabdominal ultrasound and, at an area of 0.3–0.5 cm^2^, mice were randomized to treatment arms. Pacitaxel (33 mg/kg) or saline control treatments were given by weekly IP injection for 4 weeks with 5 mice per group. Control mice served as a reference for growth kinetics but sensitivity to treatment was defined as regression below the baseline. Ultrasound measurements were taken weekly and plotted as the mean tumor area percent relative to the starting baseline size.

### Statistical analysis

Relationships between drug sensitivity and the percentage of BAK bound to antiapoptotic proteins or BCL2 family protein expression levels were analyzed using Spearman rank correlation. Differences between groups were analyzed using Student’s *t* tests with *n* − 2 degrees of freedom. Survival curve differences were analyzed using COX regression analysis. Another statistical analysis was performed with Student’s *t* tests unless otherwise mentioned. No statistical method was used to predetermine sample size. Sample sizes were chosen based on an attempt to balance the error in estimating confidence with the cost of replicating experiments. We have not estimated variation within each group.

### Reporting summary

Further information on research design is available in the Nature Research Reporting Summary linked to this article.

## Supplementary information

Supplementary Figure 1

Supplementary Figure 2

Supplementary Figure 3

Supplementary Figure 4

Supplementary Figure 5

Supplementary Figure 6

Supplementary Figure 7

Supplementary Figure 8

Supplementary Figure 9

Supplementary Figure 10

Supplementary Figure 11

Supplementary Figure Legends

Reporting Summary

Detailed Attribution of Authorship form 1

Detailed Atrribution of Authorship form 2

Change authorship form
