# Correction: Constitutive BAK/MCL1 complexes predict paclitaxel and S63845 sensitivity of ovarian cancer

**DOI:** 10.1038/s41419-021-04219-0

**Published:** 2021-10-26

**Authors:** Dongyan Liu, Xiaonan Hou, Wangyu Wu, Valentina Zafagnin, Yunjian Li, Cristina Correia, Zhiyang Zhao, Chenggang Zhao, Zhirong Liu, Tao Zhang, Zhiyou Fang, Hongzhi Wang, Chao Xu, Saravut J. Weroha, Scott H. Kaufmann, Haiming Dai

**Affiliations:** 1 Anhui Province Key Laboratory of Medical Physics and Technology, Institute of Health & Medical Technology, Hefei Institutes of Physical Science, Chinese Academy of Sciences, Hefei, 230031 China; 2 University of Science and Technology of China, Hefei, 230026 China; 3 Hefei Cancer Hospital, Chinese Academy of Sciences, Hefei, 230031 China; 4 Division of Medical Oncology, Mayo Clinic, Rochester, MN 55905 USA; 5 Second Affiliated Hospital of Anhui Medical University, Hefei, 230601 China; 6 School of Basic Medical Sciences, Anhui Medical University, Hefei, 230032 China; 7 Department of Molecular Pharmacology and Experimental Therapeutics, Mayo Clinic, Rochester, MN 55905 USA; 8 Division of Oncology Research, Mayo Clinic, Rochester, MN 55905 USA

**Keywords:** Chemotherapy, Apoptosis, Chemotherapy, Apoptosis

Correction to: *Cell Death and Disease* 10.1038/s41419-021-04073-0, published online 12 August 2021

The original version of this article unfortunately contained an error in an author name. Valentina Za**n**fagnin was missing an “n”. The original article has been corrected.

