# Supplementary figures and images for: Constitutive BAK/MCL1 complexes predict paclitaxel and S63845 sensitivity of ovarian cancer
Source: Cell Death Dis. 2021 Aug 12;12(8):789. doi: 10.1038/s41419-021-04073-0 (PMC8361168; doi:10.1038/s41419-021-04073-0)

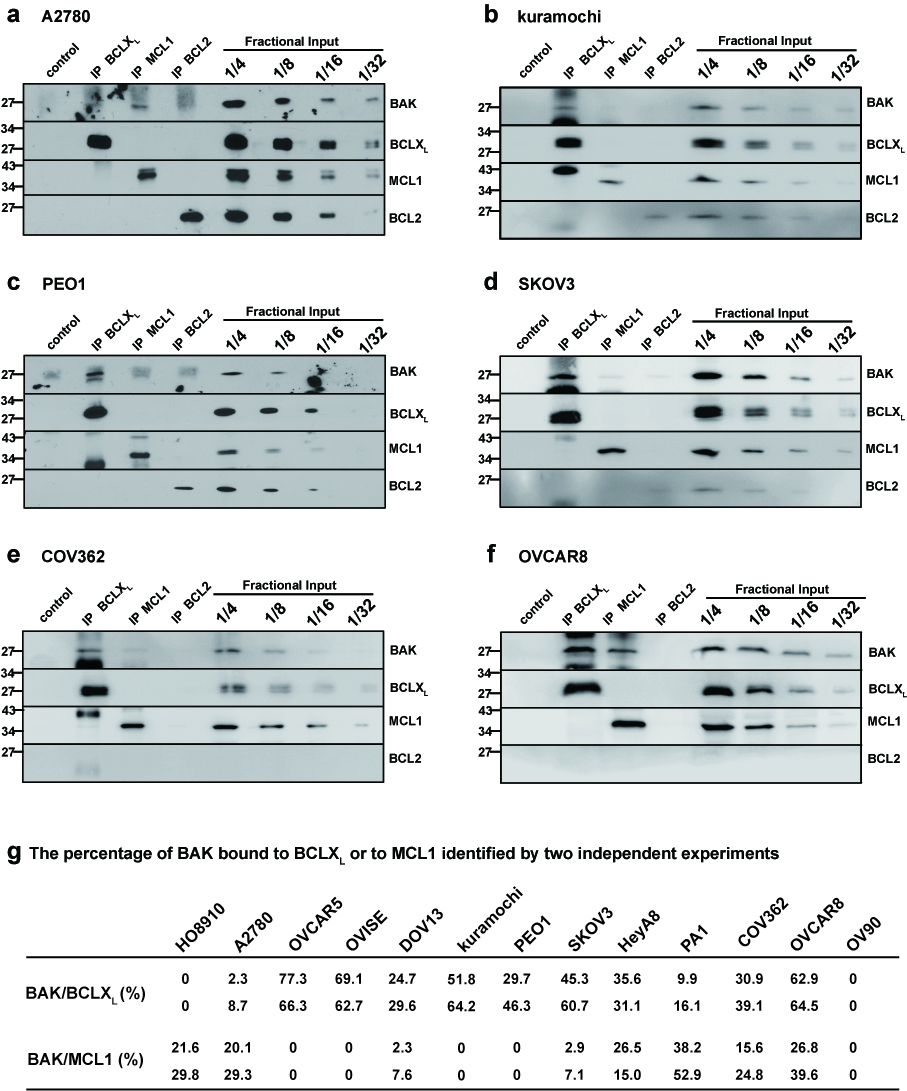

Supplement: Supplementary file 1 — Supplementary Figure 1 [file 41419_2021_4073_MOESM1_ESM.tif]

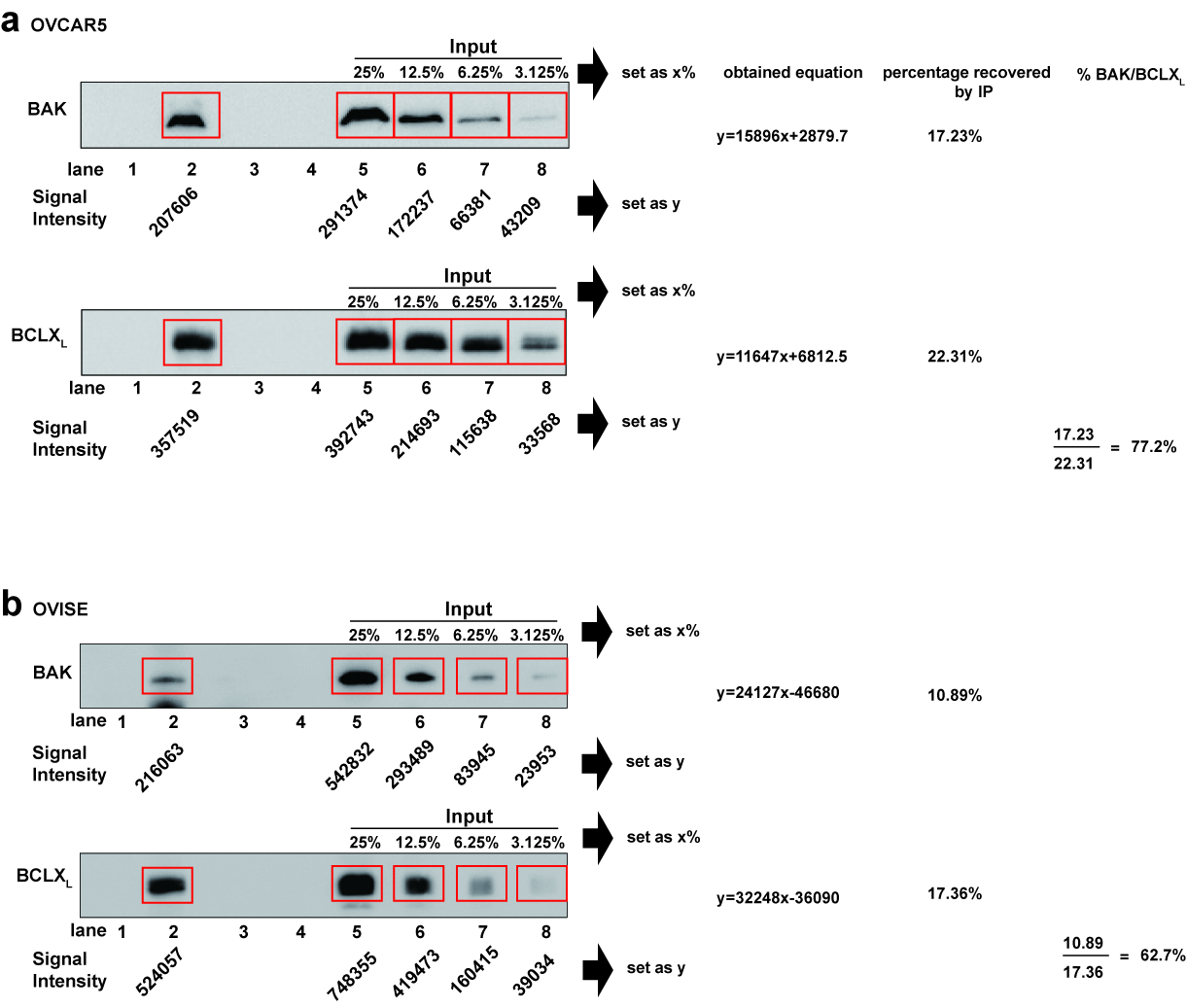

Supplement: Supplementary file 2 — Supplementary Figure 2 [file 41419_2021_4073_MOESM2_ESM.tif]

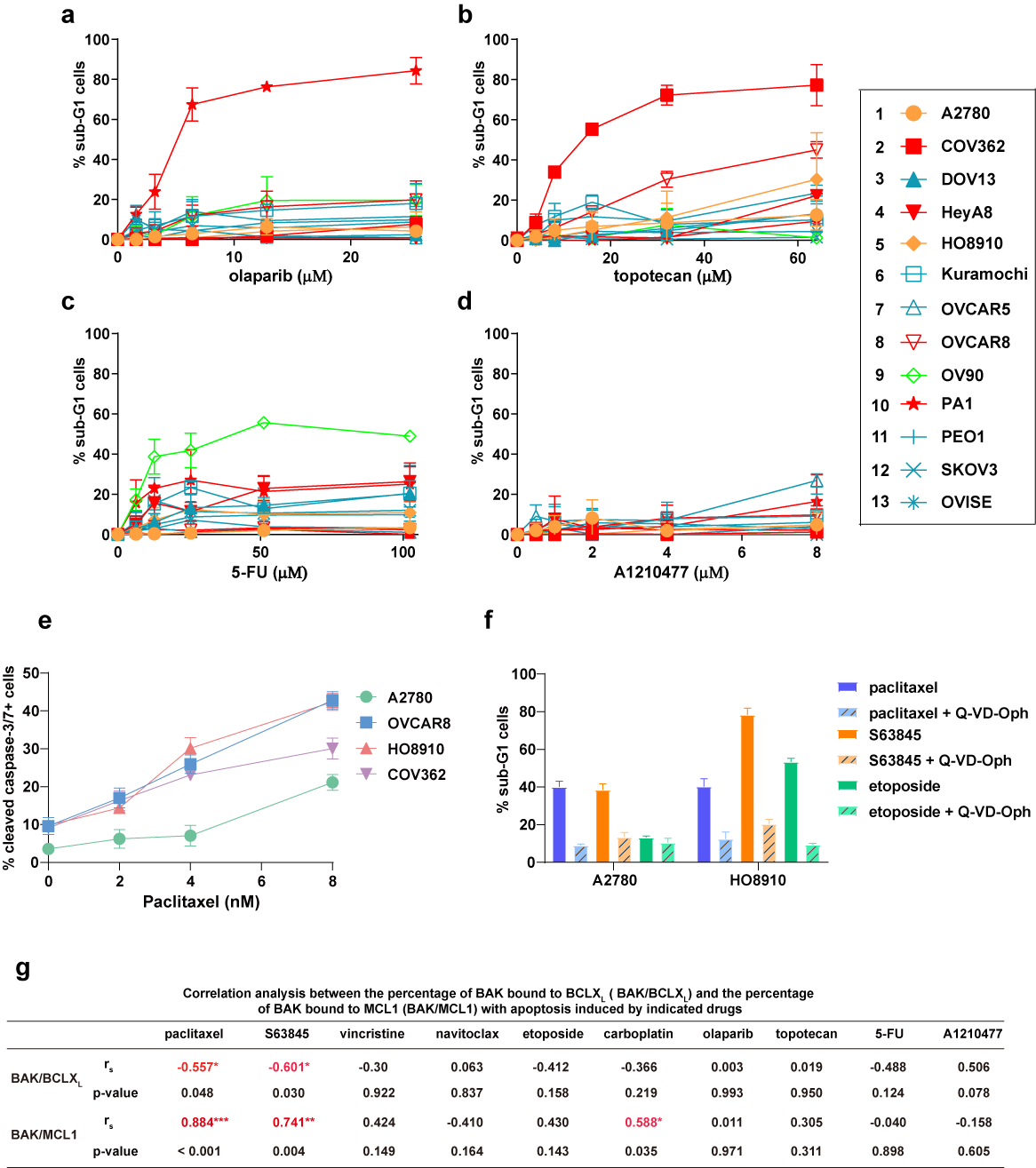

Supplement: Supplementary file 3 — Supplementary Figure 3 [file 41419_2021_4073_MOESM3_ESM.tif]

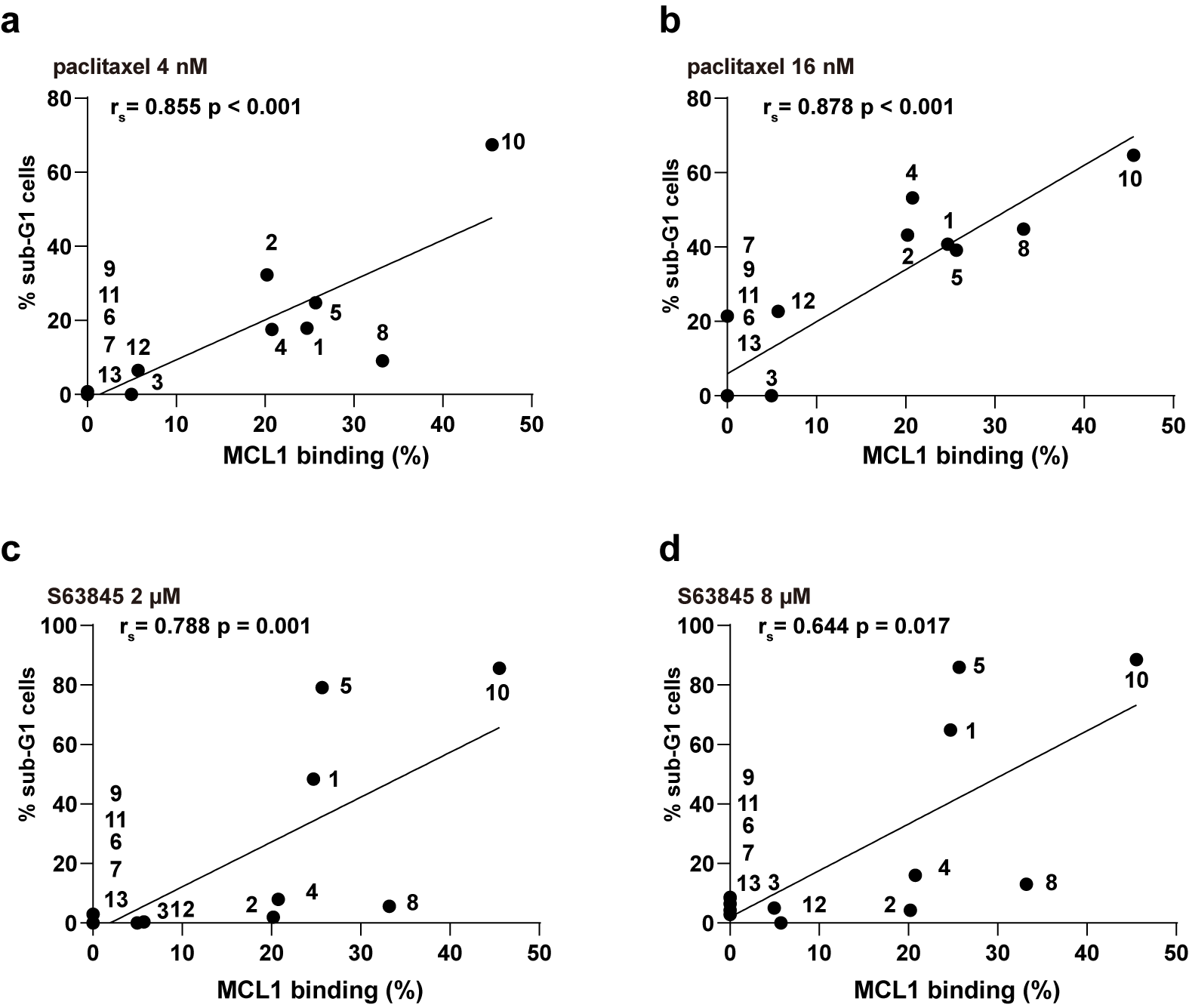

Supplement: Supplementary file 4 — Supplementary Figure 4 [file 41419_2021_4073_MOESM4_ESM.tif]

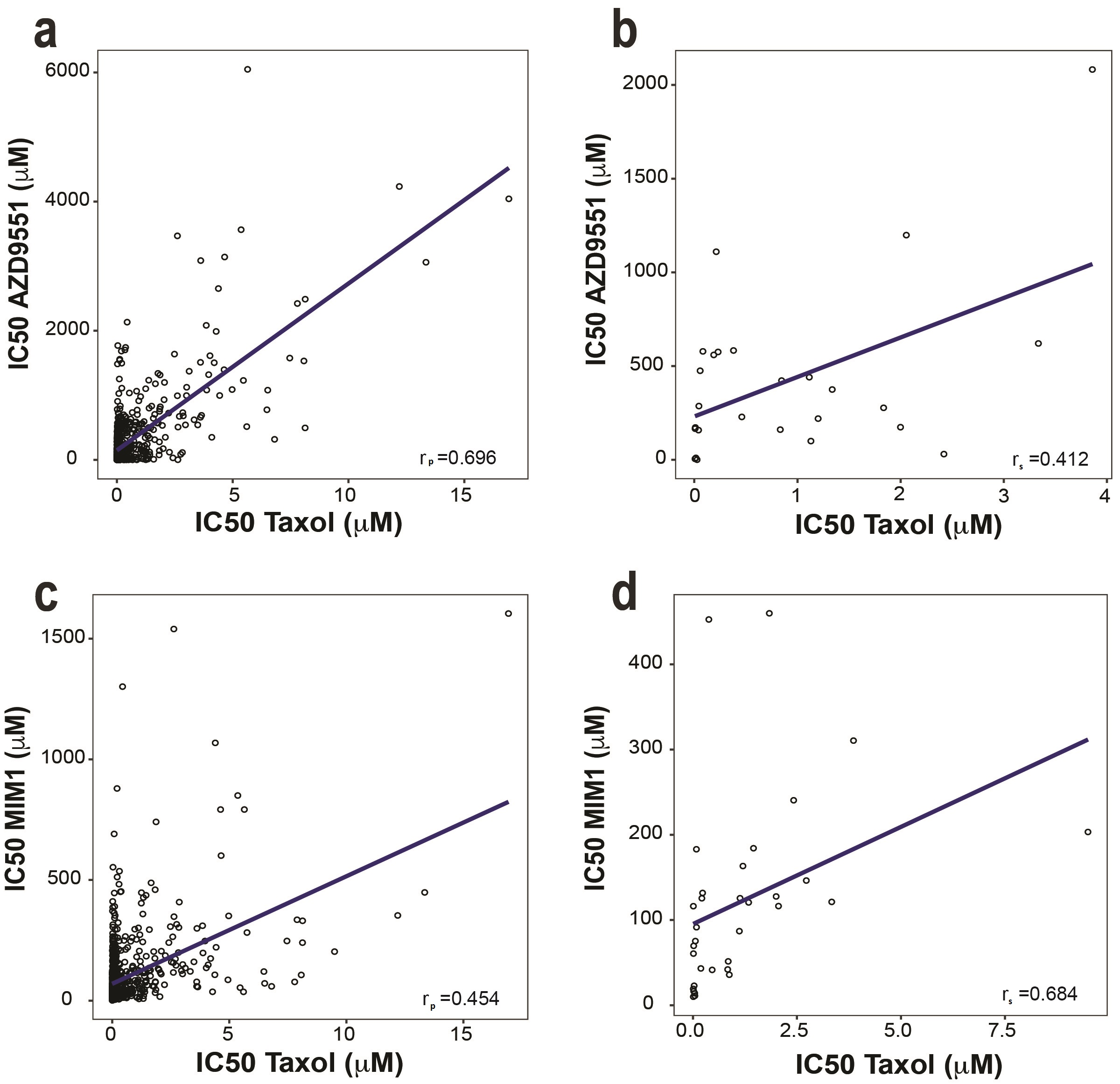

Supplement: Supplementary file 5 — Supplementary Figure 5 [file 41419_2021_4073_MOESM5_ESM.tif]

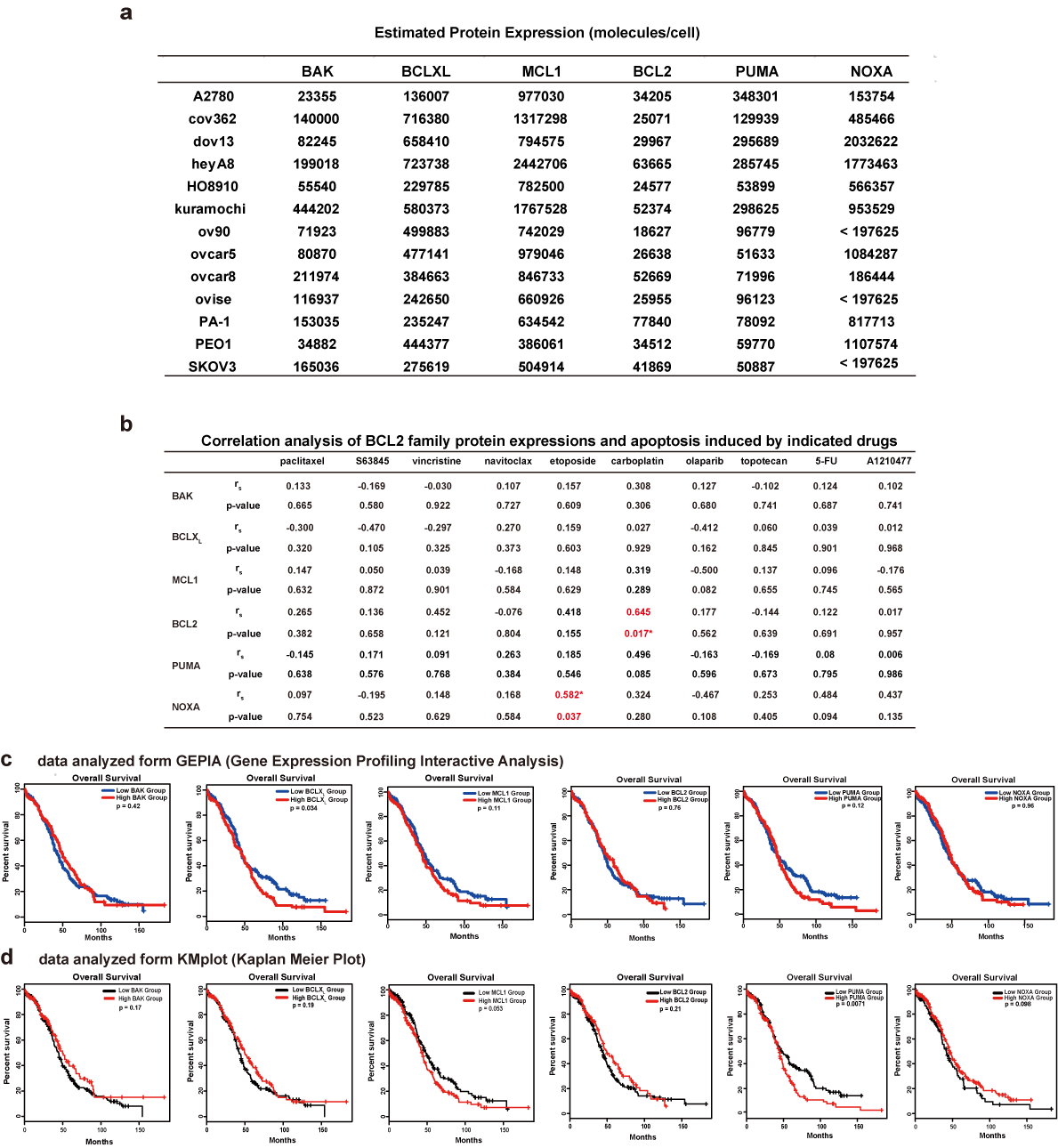

Supplement: Supplementary file 6 — Supplementary Figure 6 [file 41419_2021_4073_MOESM6_ESM.tif]

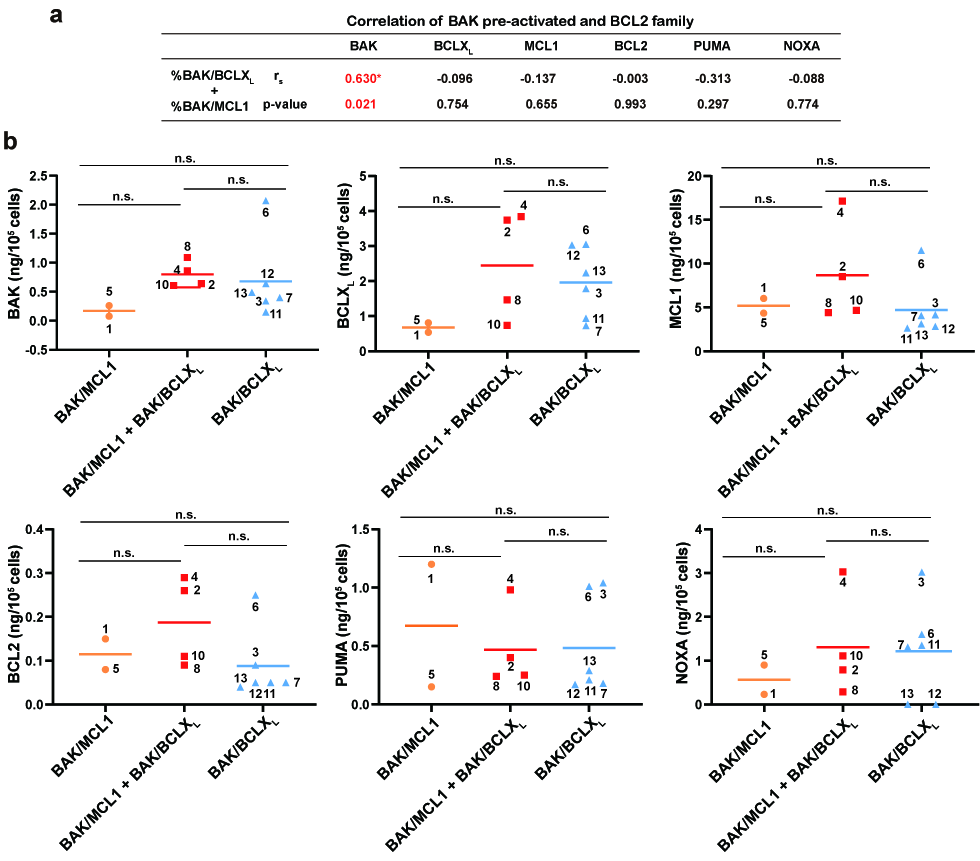

Supplement: Supplementary file 7 — Supplementary Figure 7 [file 41419_2021_4073_MOESM7_ESM.tif]

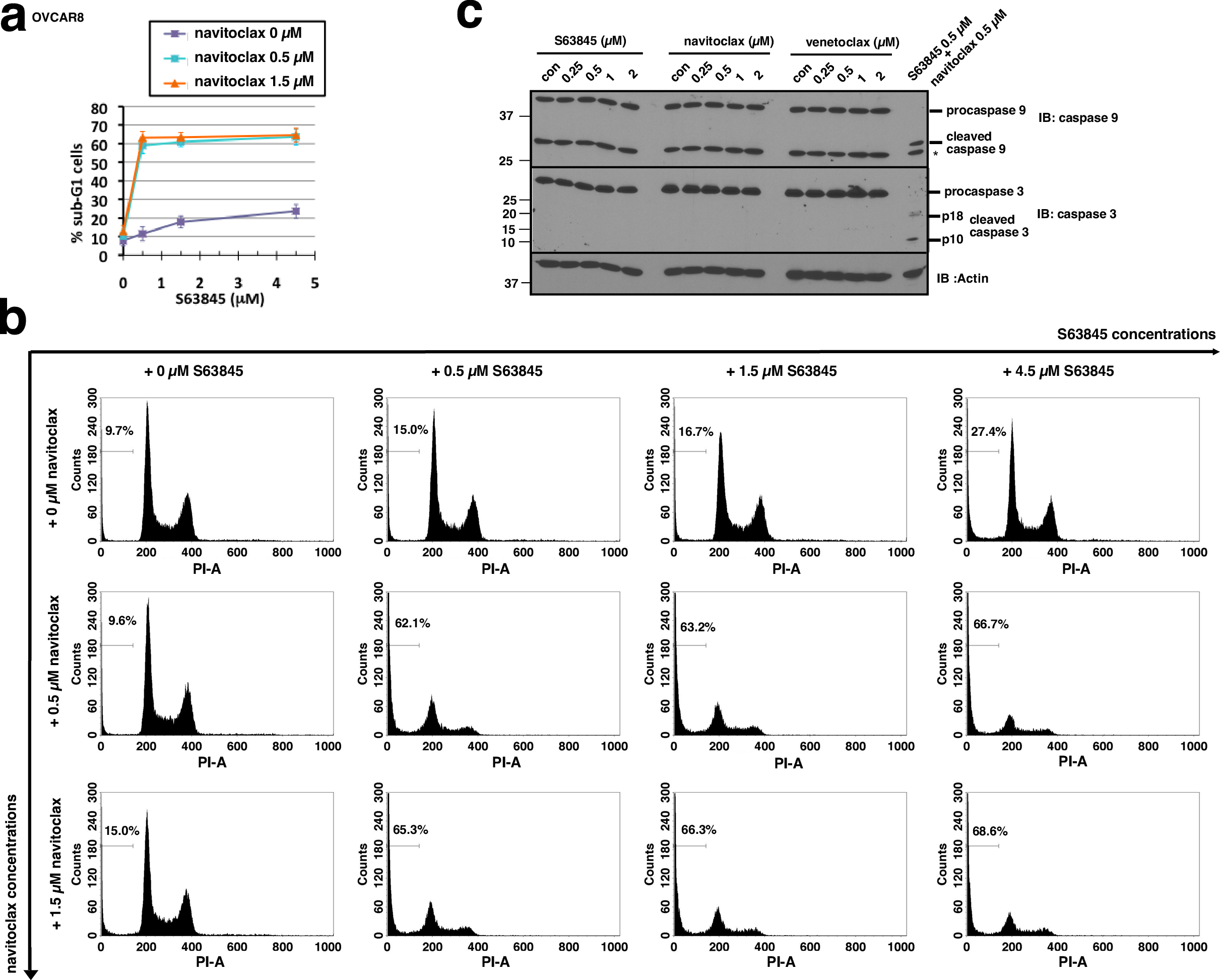

Supplement: Supplementary file 8 — Supplementary Figure 8 [file 41419_2021_4073_MOESM8_ESM.tif]

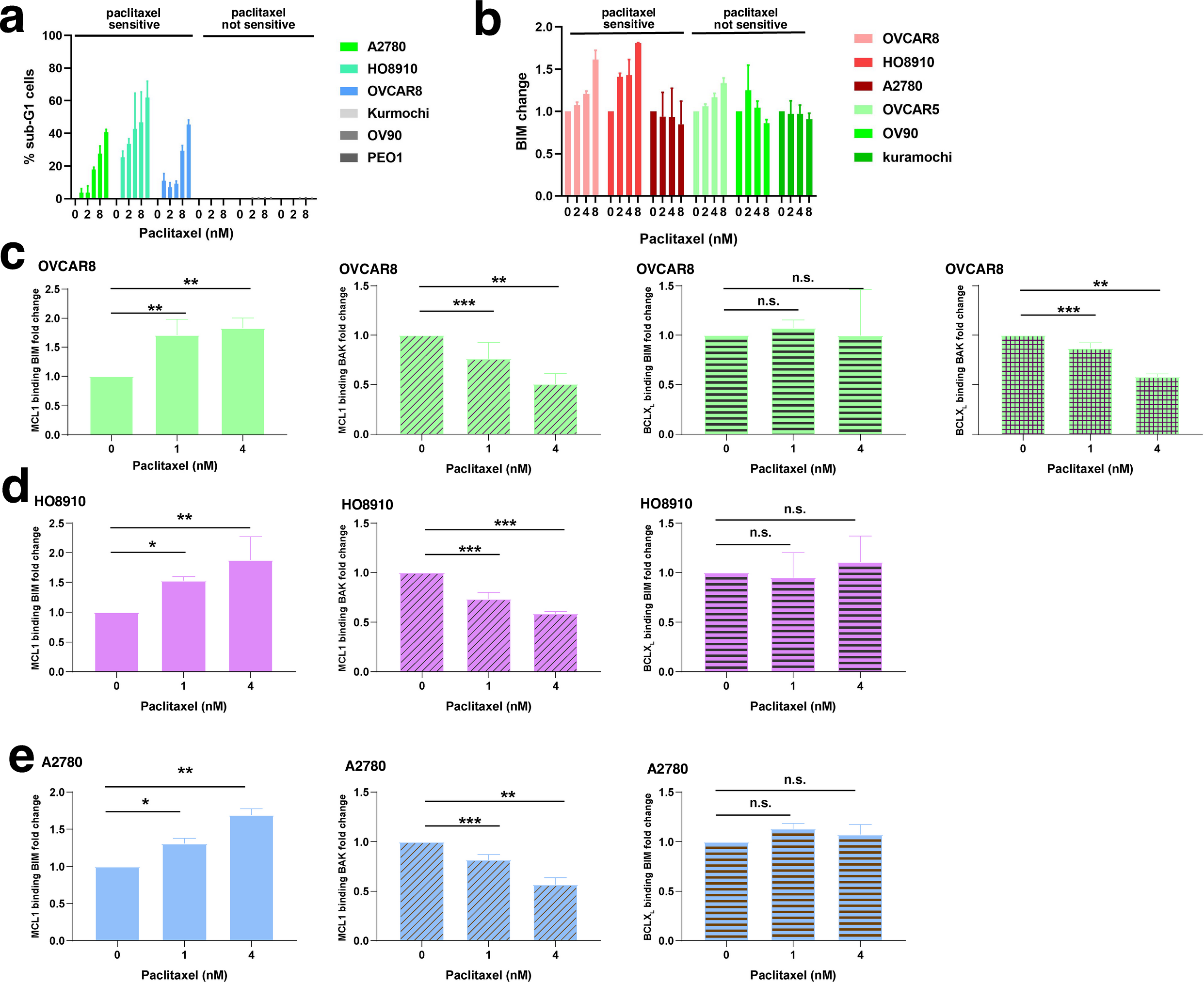

Supplement: Supplementary file 9 — Supplementary Figure 9 [file 41419_2021_4073_MOESM9_ESM.tif]

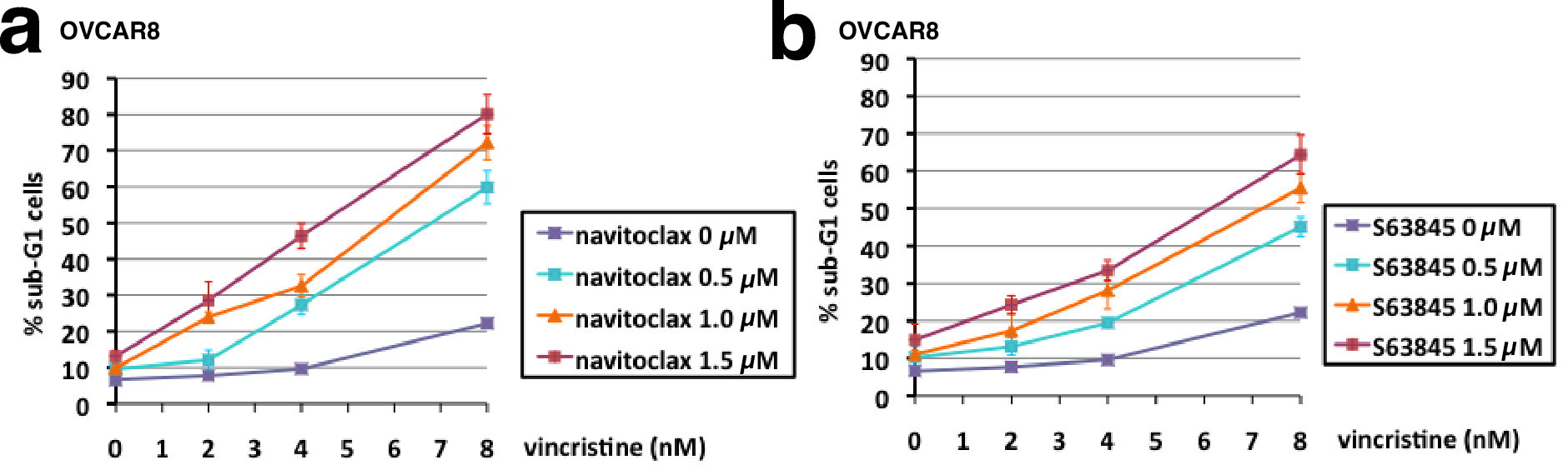

Supplement: Supplementary file 10 — Supplementary Figure 10 [file 41419_2021_4073_MOESM10_ESM.tif]

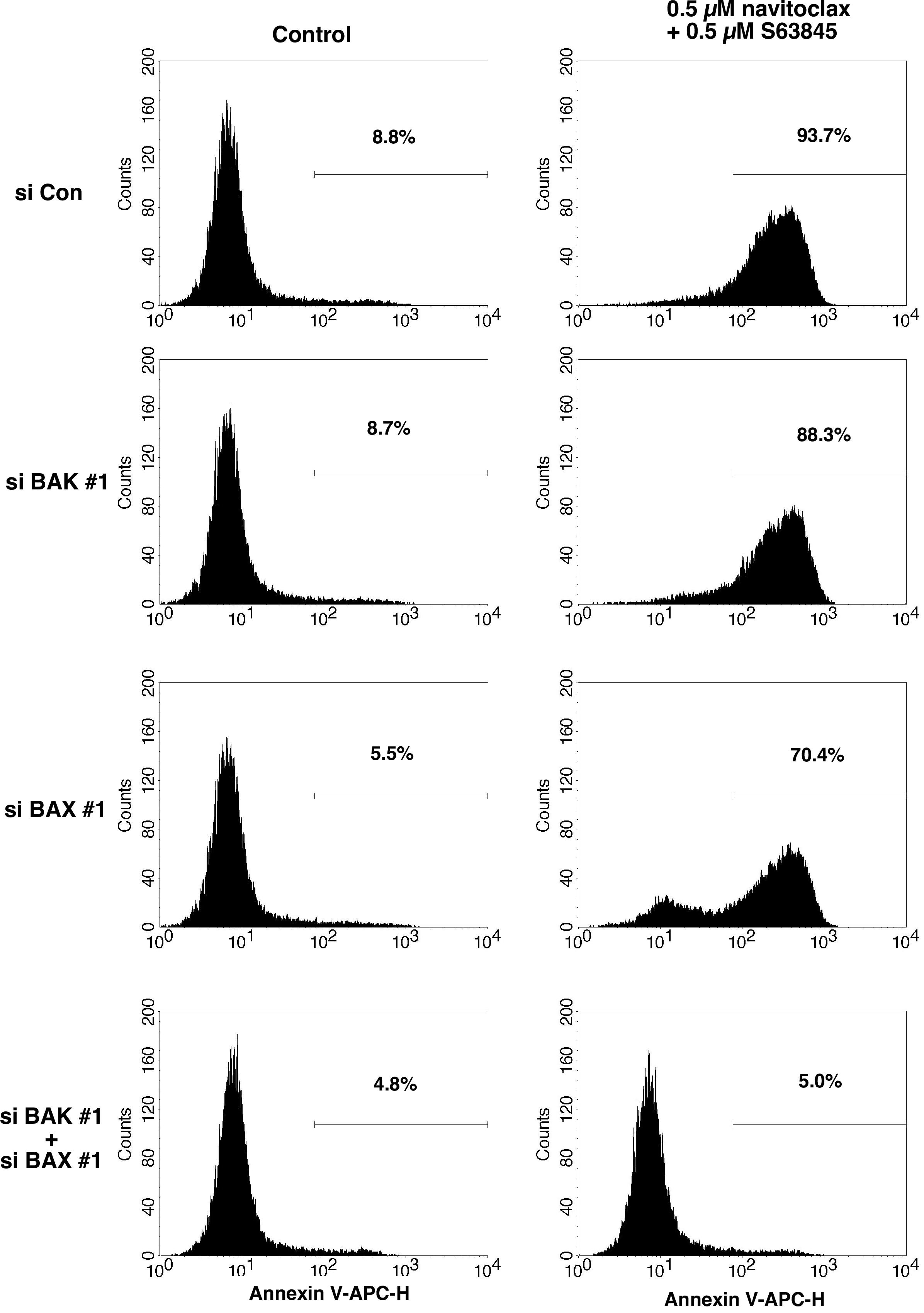

Supplement: Supplementary file 11 — Supplementary Figure 11 [file 41419_2021_4073_MOESM11_ESM.tif]
