# Supplementary material for: Constitutive BAK/MCL1 complexes predict paclitaxel and S63845 sensitivity of ovarian cancer
Source: Cell Death Dis. 2021 Aug 12;12(8):789. doi: 10.1038/s41419-021-04073-0 (PMC8361168; doi:10.1038/s41419-021-04073-0)
Supplement: Supplementary file 12 — Supplementary Figure Legends [file 41419_2021_4073_MOESM12_ESM.pdf]

SUPPLEMENTARY FIGURE LEGENDS FOR:

Constitutive BAK/MCL1 complexes predict paclitaxel and S63845 sensitivity of ovarian cancer

Dongyan Liu[1,2,3], Xiaonan Hou[4], Wangyu Wu[5], Valentina Zafagnin[4], Yunjian Li[1,6], Cristina Correia[7,8], Zhiyang Zhao[1,2,3], Chenggang Zhao[1,2,3], Zhirong Liu[1,3], Tao Zhang[5], Zhiyou Fang[1,3], Hongzhi Wang[1,3], Chao Xu[2], Saravut J. Weroha[4,7], Scott H. Kaufmann[7,8,*] and Haiming Dai[1,3,*]

Affiliations:

[1]Anhui Province Key Laboratory of Medical Physics and Technology, Institute of Health & Medical Technology, Hefei Institutes of Physical Science, Chinese Academy of Sciences, Hefei, 230031, China;
[2]University of Science and Technology of China, Hefei, 230026, China;
[3]Hefei Cancer Hospital, Chinese Academy of Sciences, Hefei, 230031, China;
[4]Division of Medical Oncology, Mayo Clinic, Rochester, MN, 55905, USA;
[5]Second Affiliated Hospital of Anhui Medical University, Hefei, 230601, China;
[6]School of Basic Medical Sciences, Anhui Medical University, Hefei, 230032, China;
[7]Department of Molecular Pharmacology and Experimental Therapeutics, Mayo Clinic, Rochester, MN, 55905, USA;
[8]Division of Oncology Research, Mayo Clinic, Rochester, MN, 55905, USA

[*], corresponding authors: Haiming Dai, E-mail: Daih@cmpt.ac.cn
or Scott H. Kaufmann, E-mail: Kaufmann.Scott@mayo.edu

SUPPLEMENTARY FIGURE LEGENDS

Supplementary Figure 1. Partially activated BAK status varies among ovarian cancer cell lines. a-f CHAPS lysates of A2780 (**a**), Kuramochi (**b**), PEO1 (**c**), SKOV3 (**d**), COV362 (**e**), and OVCAR8 (**f**) were immunoprecipitated (IP) with antibodies to $BCLX_L$, MCL1, or BCL2, and compared with serial dilutions of the input. IPs with normal rabbit IgG served as negative controls. **g** The percentages of BAK bound to $BCLX_L$ (BAK/$BCLX_L$) or to MCL1 (BAK/MCL1) in 13 ovarian cancer cell lines were identified by IP assay from two independent experiments.

Supplementary Figure 2. Calculation of the percentage of BAK bound to $BCLX_L$ or to MCL1. a-b After the signal intensities of the indicated bands on the immuno-precipitation (IP) blots from OVCAR5 (**a**) or OVISE (**b**) were acquired by Image J, an best fit line between the signal intensity (y) and the percentage of Input (x) was obtained. The percentage of BAK recovered by IP was then estimated. The percentages of BAK bound to $BCLX_L$ (%BAK/$BCLX_L$) and to MCL1 (%BAK/MCL1) were calculated accordingly.

Supplementary Figure 3. Pre-formed BAK/MCL1 complexes predict cancer cell sensitivity to paclitaxel and S63845

a-d After the cell lines were treated with olaparib (**a**), topotecan (**b**), 5-FU (**c**), or A1210477 (**d**) for 48 h, the percentages of sub-G1 cells were assessed by flow cytometry (n=3~5 independent experiments, Means ± S.D.). Cells with BAK status classified as BAK/MCL1, BAK/$BCLX_L$, BAK/MCL1+BAK/$BCLX_L$, and none are indicated with orange, blue, red and green, respectively. **e** After the cell lines were treated with indicated concentrations of paclitaxel for 48 h, the percentage of cleaved caspase-3/7+ cells were assessed by flow cytometry (n=3 independent experiments, Means ± S.D.) **f** After the cell lines were treated with paclitaxel (8 nM), S63845 (4 μM) or etoposide (7.5 μM) with or without 10 μM Q-VD-OPh for 48 h, the percentage of sub-G1 cells were assessed (n=3 independent experiments, Means ± S.D.). **g** Spearman rank correlation was used to analyze the correlation between the percentage of BAK bound to $BCLX_L$ (BAK/$BCLX_L$) or the percentage of BAK bound to MCL1 (BAK/MCL1) with apoptosis induced by the indicated drugs.

Supplementary Figure 4. The presence of BAK/MCL1 complexes correlated with cell death induced by paclitaxel and S63845. a-d The correlations of BAK/MCL1 complexes and cell death induced by paclitaxel 4 nM (**a**), paclitaxel 16 nM (**b**), S63845 2 μM (**c**), and S63845 8 μM (**d**) were analyzed using Spearman rank correlation, respectively.

Supplementary Figure 5. Paclitaxel sensitivity correlates with sensitivities to two different MCL1 inhibitors in the Genomics of Drug Sensitivity in Cancer database. Cells were exposed to the indicated drugs for 48 h and assayed for viability by Promega CellTiter-Glo. **a-b** IC_{50} to paclitaxel (μM) and IC_{50} to AZD5991 (μM) were analyzed through scatter plots across all cell lines (**a,** $r_p = 0.696$, $p < 2.97e-10$) or ovarian cell lines (**b,** $r_s = 0.412$, $p = 0.04$) in the Genomics of Drug Sensitivity in Cancer database (https://www.cancerrxgene.org). **c-d** IC_{50} to paclitaxel (μM) and IC_{50} to MIM1 (μM) were analyzed through scatter plots across all cell lines (**c,** $r_p = 0.454$, $p < 2.2e-16$) or ovarian cell lines (**d,** $r_s = 0.684$, $p = 1.88e-5$).

Supplementary Figure 6. Expression of BCL2 family members at the protein or mRNA levels does not predict paclitaxel sensitivity in ovarian cancer. a BCL2 family proteins expression in untreated ovarian cancer cell lines. Data represents one from two independent experiments. **b** Spearman rank correlation was used to analyze the correlation between each of the BCL2 family protein levels with apoptosis induced by the indicated drugs. **c-d** Ovarian cancer patients from GEPIA (**c**), and KMplot database (**d**) were grouped according to mRNA expression levels of BAK, $BCLX_L$, MCL1, BCL2, PUMA, and NOXA, respectively, and the overall survival was analyzed.

Supplementary Figure 7. Constitutive binding of BAK to $BCLX_L$ or MCL1 does not correlate with any single BCL2 family protein. a Spearman correlation analysis between protein levels (ng/10^5 cells) of BAK, $BCLX_L$, MCL1, BCL2, PUMA or NOXA and %BAK/$BCLX_L$ + % BAK/MCL1. **b** After ovarian cancer cells were divided into BAK/MCL1, BAK/MCL1+BAK/$BCLX_L$, and BAK/$BCLX_L$, the levels of BAK, $BCLX_L$, MCL1, BCL2, PUMA or NOXA protein were compared between each group. n.s., p>0.05.

Supplementary Figure 8. S63845 in combination with navitoclax induces cell death in OVCAR8 cells. a-b After OVCAR8 cells were treated with the indicated concentrations of S63845 combined with navitoclax for 48 h, the percentages of sub-G1 cells were detected by flow cytometry. (**a**, n=3 independent experiments, mean ± S.D.) **b**, a representative experiment was shown. **c** After OVCAR8 cells were treated with the indicated concentrations of S63845, navitoclax, or venetoclax, whole cell lysates were blotted with the indicated antibodies.

Supplementary Figure 9. Paclitaxel-induced BIM preferentially bind to MCL1. a Indication of the percentage of sub-G1 cells of 3 paclitaxel sensitive cell lines and 3 paclitaxel not sensitive cell lines after 48 h of paclitaxel treatments. **b**, the fold change of BIM protein after paclitaxel treatment estimated by western blot (n=3 independent experiments, mean ± S.D.) . **c-e**, After OVCAR8 (**c**), HO8910 (**d**), or A2780 (**e**) cells were treated with indicated concentrations of paclitaxel, in the presence of Q-VD-OPh for 48 h, CHAPS lysates were immunoprecipitated with antibodies to $BCLX_L$ or MCL1. The endogenous MCL1 binding BIM, MCL1 binding BAK, $BCLX_L$ binding BIM, or $BCLX_L$ binding BAK were analyzed respectively, comparing paclitaxel treated bands to non-treated bands (n=3 independent experiments, mean ± S.D.).

Supplementary Figure 10. Navitoclax or S63845 in combination with paclitaxel induces cell death. a-b, After OVCAR8 cells were treated with indicated concentrations of navitoclax (**a**) or S63845 (**b**) in combination with vincristine for 48 h, the percentages of sub-G1 cells were assayed by flow cytometry.

Supplementary Figure 11. BAK siRNA and BAX siRNA together inhibit cell death induced by navitoclax + S63845 in OVCAR8 cells. 24 h after OVCAR8 cells were transfected with the indicated siRNA, cells were treated with indicated concentrations of S63845 and navitoclax for 48 h and assayed for Annexin V binding by flow cytometry. A representative experiment is shown.
