# Supplementary material for: Constitutive BAK/MCL1 complexes predict paclitaxel and S63845 sensitivity of ovarian cancer
Source: Cell Death Dis. 2021 Aug 12;12(8):789. doi: 10.1038/s41419-021-04073-0 (PMC8361168; doi:10.1038/s41419-021-04073-0)
Supplement: Supplementary file 14 — Detailed Attribution of Authorship form 1 [file 41419_2021_4073_MOESM14_ESM.pdf]

DECLARATION OF CONTRIBUTIONS TO ARTICLE

ADMC

Manuscript Number: CDDIS-20-4580

Journal Name: *Cell Death & Disease* (the 'Journal')

Proposed Title of the Contribution: Contitutive BAK/MCL1 complexes predict paclitaxel and S63845 sensitivity of ovarian cancer (the 'Contribution)

Author(s): Dongyan Liu, Xiaonan Hou, Wangyu Wu, Valentina Zafagnin, Yunjian Li, Cristina Correia, Zhiyang Zhao, Chenggang Zhao, Zhirong Liu, Tao Zhang, Zhiyou Fang, Hongzhi Wang, Chao Xu, Saravut J., Weroha, Scott H. Kaufmann, Haiming Dai (the 'Authors')

For all *CDDis* articles, each person named as an author in the published version must be able to show he or she has contributed substantially to the article.

Authorship credit should be based on 1) substantial contributions to conception and design, acquisition of data, or analysis and interpretation of data; 2) drafting the article or revising it critically for important intellectual content; and 3) final approval of the version to be published. Authors should meet conditions 1, 2 and 3.

Any person who cannot be shown to have made a substantial contribution to the article cannot be listed as an author in the final version. The name of any person who is deemed to have made a minor contribution can, however, appear in the Acknowledgments section of the article.

Please complete the table below to indicate the contributions of all named authors to the manuscript.

Author Full Name:	Specification of Contribution to the Manuscript:
Dongyan Liu	Experiments and data analysis, drafting the article, final approval of the version to be published
Xiaonan Hou	Experiments and data analysis, final approval of the version to be published
Wangyu Wu	Experiments and data analysis, final approval of the version to be published
Valentina Zafagnin	Experiments and data analysis, final approval of the version to be published
Yunjian Li	Experiments and data analysis, final approval of the version to be published
Cristina Correia	Experiments and data analysis, final approval of the version to be published
Zhiyang Zhao	Experiments and data analysis, final approval of the version to be published
Chenggang Zhao	Experiments and data analysis, final approval of the version to be published
Zhirong Liu	Experiments and data analysis, final approval of the version to be published
Tao Zhang	Experiments and data analysis, final approval of the version to be published
Zhiyou Fang	Experiments and data analysis, final approval of the version to be published
Hongzhi Wang	Experiments and data analysis, final approval of the version to be published
Chao Xu	Experiments and data analysis, final approval of the version to be published

Please complete the table below to indicate the contributions of all named authors to the figures.

Figure 1:

Dongyan Liu, Chenggang Zhao, Zhiyang Zhao, Zhiyou Fang

Figure 2:

Dongyan Liu, Yunjian Li, Tao Zhang

Figure 3:

Xiaonan Hou, Valentina Zafagnin, Saravut J. Weroha, Scott H. Kaufmann, Haiming Dai

Figure 4:

Dongyan Liu

Figure 5:

Dongyan Liu, Yunjian Li, Haiming Dai

Figure 6:

Dongyan Liu, Haiming Dai

Signed for and on behalf of the Author(s):	Print Name:	Date:
Haiming Dai	Haiming Dai	03/08/2021
