# Supplementary material for: Constitutive BAK/MCL1 complexes predict paclitaxel and S63845 sensitivity of ovarian cancer
Source: Cell Death Dis. 2021 Aug 12;12(8):789. doi: 10.1038/s41419-021-04073-0 (PMC8361168; doi:10.1038/s41419-021-04073-0)
Supplement: Supplementary file 15 — Detailed Atrribution of Authorship form 2 [file 41419_2021_4073_MOESM15_ESM.pdf]

DECLARATION OF CONTRIBUTIONS TO ARTICLE

ADMC

Manuscript Number:	Journal Name:	
CDDIS-20-4580 form 2	*Cell Death & Disease*	(the 'Journal')

Proposed Title of the Contribution:

Contitutive BAK/MCL1 complexes predict paclitaxel and S63845 sensitivity of ovarian cancer (the 'Contribution)

Author(s):

Dongyan Liu, Xiaonan Hou, Wangyu Wu, Valentina Zafagnin, Yunjian Li, Cristina Correia, Zhiyang Zhao, Chenggang Zhao, Zhirong Liu, Tao Zhang, Zhiyou Fang, Hongzhi Wang, Chao Xu, Saravut J., Weroha, Scott H. Kaufmann, Haiming Dai (the 'Authors')

For all *CDDis* articles, each person named as an author in the published version must be able to show he or she has contributed substantially to the article.

Authorship credit should be based on 1) substantial contributions to conception and design, acquisition of data, or analysis and interpretation of data; 2) drafting the article or revising it critically for important intellectual content; and 3) final approval of the version to be published. Authors should meet conditions 1, 2 and 3.

Any person who cannot be shown to have made a substantial contribution to the article cannot be listed as an author in the final version. The name of any person who is deemed to have made a minor contribution can, however, appear in the Acknowledgments section of the article.

Please complete the table below to indicate the contributions of all named authors to the manuscript.

Author Full Name:	Specification of Contribution to the Manuscript:
Saravut J. Weroha	Experiments and data analysis, final approval of the version to be published
Scott H. Kaufmann	Concept and design, experiments and data analysis, revising the manuscript, final approval of the version to be published
Haiming Dai	Concept and design, experiments and data analysis, drafting and revising the manuscript, final approval of the version to be published

Please complete the table below to indicate the contributions of all named authors to the figures.

Figure 7:

Dongyan Liu, Wangyu Wu, Tao Zhang

Figure 8:

Figure 9:

Figure 10:

Figure 11:

Figure 12:

Signed for and on behalf of the Author(s):	Print Name:	Date:
	Haiming Dai	03/08/2021
