# Supplementary material for: Constitutive BAK/MCL1 complexes predict paclitaxel and S63845 sensitivity of ovarian cancer
Source: Cell Death Dis. 2021 Aug 12;12(8):789. doi: 10.1038/s41419-021-04073-0 (PMC8361168; doi:10.1038/s41419-021-04073-0)
Supplement: Supplementary file 16 — Change authorship form [file 41419_2021_4073_MOESM16_ESM.pdf]

SPRINGER NATURE

Change of authorship request form - Journals (pre-acceptance)

Important information. Please read.

- This form should be used by authors to request any change in authorship (adding/deleting authors) including changes in corresponding authors. This form should not be used for name changes. Please fully complete all sections. Use black ink and block capitals and provide each author's full name with the given name first followed by the family name.
- By signing this declaration, all authors guarantee that the order of the authors are in accordance with their scientific contribution, if applicable as different conventions apply per discipline, and that only authors have been added who made a meaningful contribution to the work.
- Please note, in author collaborations where there is formal agreement for representing the collaboration, it is sufficient for the representative or legal guarantor (usually the corresponding author) to complete and sign the Authorship Change Form on behalf of all authors, **next to the added/removed author(s)**. **(Complete Section 3, followed by Section 6.)**
  In author collaborations where there is no formal agreement for representing the collaboration and **there are more than 10 authors**, one may sign for all, provided the signer appends correspondence that attests that each of the authors have agreed to the change **and the added/removed authors sign the form**. **(Complete Section 3, followed by Section 6.)**
- Please note, we cannot investigate or mediate any authorship disputes. If you are unable to obtain agreement from all authors (including those who you wish to be removed) you must refer the matter to your institution(s) for investigation. Please inform us if you need to do this.
- If you are not able to return a fully completed form within **30 days** of the date that it was sent to the author requesting the change, we may have to withdraw your manuscript. We cannot publish manuscripts where authorship has not been agreed by all authors (including those who have been removed).
- Incomplete forms will be rejected.
- Please return/upload this form, fully completed, to the Journals Editorial Office. The Journal and/or Publisher will consider the information you have provided to decide whether to approve the proposed change in authorship. We may decide to contact your institution for more information or undertake a further investigation, if appropriate, before making a final decision.

SPRINGER NATURE

Change of authorship request form - Journals (pre-acceptance)

Section 1: Please provide the current title of manuscript

Manuscript ID no.: CDDIS-20-4580

Title: Constitutive BAK/MCL1 complexes predict paclitaxel and S63845 sensitivity of ovarian cancer

Section 2: Please provide the previous authorship, in the order shown on the manuscript before the changes were introduced. Please indicate the corresponding author by adding (CA) behind the name.

	First name(s)	Family name	ORCID or SCOPUS id, if available
1st author	Dongyan	Liu	
2nd author	Xiaonan	Hou	
3rd author	Wangyu	Wu	
4th author	Valentina	Zanfagnin	
5th author	Yunjian	Li	
6th author	Zhiyang	Zhao	
7th author	Chengang	Zhao	
8th author	Zhirong	Liu	
9th author	Tao	Zhang	
10th author	Zhiyou	Fang	

Please use an additional sheet if there are more than 10 authors.

SPRINGER NATURE Springer Nature is one of the world's leading global research, educational and professional publishers, created in May 2015 through the combination of Nature Publishing Group, Palgrave Macmillan, Macmillan Education and Springer Science+Business Media.

SPRINGER NATURE

Change of authorship request form - Journals (pre-acceptance)

Section 1: Please provide the current title of manuscript

Manuscript ID no.: CDDIS-20-4580

Title: Constitutive BAK/MCL1 complexes predict paclitaxel and S63845 sensitivity of ovarian cancer

Section 2: Please provide the previous authorship, in the order shown on the manuscript before the changes were introduced. Please indicate the corresponding author by adding (CA) behind the name.

	First name(s)	Family name	ORCID or SCOPUS id, if available
1st author	Hongzhi	Wang	
2nd author	Chao	Xu	
3rd author	Saravut	Weroha	
4th author	Scott	Kaufmann	
5th author	Haiming	Dai	
6th author			
7th author			
8th author			
9th author			
10th author			

Please use an additional sheet if there are more than 10 authors.

SPRINGER NATURE Springer Nature is one of the world's leading global research, educational and professional publishers, created in May 2015 through the combination of Nature Publishing Group, Palgrave Macmillan, Macmillan Education and Springer Science+Business Media.

SPRINGER NATURE

Change of authorship request form - Journals (pre-acceptance

Section 3: Please provide a justification for change. Please use this section to explain your reasons for changing the authorship of your manuscript, e.g. what necessitated the change in authorship? Please refer to the (journal) policy pages for more information about authorship. Please explain why omitted authors were not originally included and/or why authors were removed on the submitted manuscript.

We have included Cristina Correia in the new authorship list. Because when we revised the manuscript, we tried to include a bioinformatic analysis of the correlation between drug sensitivities to taxol and drug sensitivities to MCL1 inhibitors in the public database. Cristina Correia is an expert in this area, and she did the search of the database and the analysis. Moreover, the data she obatined further supported our results and was included in our manuscript.

Section 4: Proposed new authorship. Please provide your new authorship list in the order you would like it to appear on the manuscript. Please indicate the corresponding author by adding (CA) behind the name. <u>If the Corresponding Author has changed, please indicate the reason under section 3.</u>

	First name(s)	Family name (this name will appear in full on the final publication and will be searchable in various abstract and indexing databases)	Affiliated institute	E-mail address
1st author	Dongyan	Liu	Hefei Institutes of Physical Science, CAS	liudy209@163.com
2nd author	Xiaonan	Hou	Mayo Clinic	Hou.xiaonan@mayo.edu
3rd author	Wangyu	Wu	Second Affiliated Hospital of Anhui Medical University	249697251@qq.com
4th author	Valentina	Zanfagnin	Mayo Clinic	Zanfagnin.valentina@mayo.edu
5th author	Yunjian	Li	Hefei Institutes of Physical Science, CAS	1109619197@qq.com
6th author	Cristina	Correia	Mayo Clinic	correia.cristina@mayo.edu
7th author	Zhiyang	Zhao	Hefei Institutes of Physical Science, CAS	zzhiyang@mail.ustc.edu.cn
8th author	Chengang	Zhao	Hefei Institutes of Physical Science, CAS	352782963@qq.com
9th author	Zhirong	Liu	Hefei Institutes of Physical Science, CAS	458727657@qq.com
10th author	Tao	Zhang	Second Affiliated Hospital of Anhui Medical University	ayzt_urology@163.com

Please use an additional sheet if there are more than 10 authors.

SPRINGER NATURE

Change of authorship request form - Journals (pre-acceptance)

Section 3: Please provide a justification for change. Please use this section to explain your reasons for changing the authorship of your manuscript, e.g. what necessitated the change in authorship? Please refer to the (journal) policy pages for more information about authorship. Please explain why omitted authors were not originally included and/or why authors were removed on the submitted manuscript.

Section 4: Proposed new authorship. Please provide your new authorship list in the order you would like it to appear on the manuscript. Please indicate the corresponding author by adding (CA) behind the name. <u>If the Corresponding Author has changed, please indicate the reason under section 3</u>.

	First name(s)	Family name (this name will appear in full on the final publication and will be searchable in various abstract and indexing databases)	Affiliated institute	E-mail address
1st author	Zhiyou	Fang	Hefei Institutes of Physical Science, CAS	z.fang@cmpt.ac.cn
2nd author	Hongzhi	Wang	Hefei Institutes of Physical Science, CAS	wanghz@hfcas.ac.cn
3rd author	Chao	Xu	University of Science and Technology of China	xuchaor@ustc.edu.cn
4th author	Saravut	Weroha	Mayo Clinic	Weroha.Saravut@mayo.edu
5th author	Scott	Kaufmann	Mayo Clinic	kaufmann.scott@mayo.edu
6th author	Haiming	Dai	Hefei Institutes of Physical Science, CAS	daih@cmpt.ac.cn
7th author				
8th author				
9th author				
10th author				

Please use an additional sheet if there are more than 10 authors.

SPRINGER NATURE Springer Nature is one of the world's leading global research, educational and professional publishers, created in May 2015 through the combination of Nature Publishing Group, Palgrave Macmillan, Macmillan Education and Springer Science+Business Media.

SPRINGER NATURE Change of authorship request form - Journals (pre-acceptance)

Section 5: Author contribution, Acknowledgement and Disclosures. Please use this section to provide a new disclosure statement and, if appropriate, acknowledge any contributors who have been removed as authors and ensure you state what contribution any new authors made (if applicable per the journal or book (series) policy). Please ensure these are updated in your manuscript - after approval of the change(s) - as our production department will not transfer the information in this form to your manuscript.

New acknowledgements:

We thank for David Huang from WEHI for the antibody.

New Disclosures (financial and non-financial interests, funding):

The authors report no conflict of interest.

New Author Contributions statement (if applicable per the journal policy):

H.D. and S.H.K. conceived and designed the study. D.L., X.H., W.W., V.Z., Y.L., C.C., Z.Z., C.Z., Z.L., T.Z., Z.F., H.W., C.X., S.J.W. and H.D. performed the experiments. H.D., S.H.K., and D.L wrote the manuscript. All authors reviewed the manuscript.

State 'Not applicable' if there are no new authors.

SPRINGER NATURE   Change of authorship request form - Journals (pre-acceptance)

Section 6: Declaration of agreement. *All* authors, unchanged, new and removed *must* sign this declaration.
(NB: Please print the form, (docu)-sign and return/upload a scanned copy. Please note that signatures that have been inserted as an image file are acceptable as long as it is handwritten. Typed names in the signature box are unacceptable.) *** Please delete as appropriate. Delete all of the bold if you were on the original authorship list and are remaining as an author.**

	First name	Family name		Signature	Date
1st author	Dongyan	Liu	I agree to the proposed new authorship shown in section 4 **/and the addition/removal*of my name to the authorship list** /and the proposed change in corresponding author	Dongyan Liu	04 09 2021
2nd author	Xiaonan	Hou	I agree to the proposed new authorship shown in section 4 **/and the addition/removal*of my name to the authorship list** /and the proposed change in corresponding author	[illegible]	04 08 2021
3rd author	Wangyu	Wu	I agree to the proposed new authorship shown in section 4 **/and the addition/removal*of my name to the authorship list** /and the proposed change in corresponding author	Wangyu Wu	04 11 2021
4th authors	Valentina	Za~~[illegible]~~gnin ZANFAGNIN	I agree to the proposed new authorship shown in section 4 **/and the addition/removal*of my name to the authorship list** /and the proposed change in corresponding author	Valentina Zanfagnin	4/12/21
5th author	Yunjian	Li	I agree to the proposed new authorship shown in section 4 **/and the addition/removal*of my name to the authorship list** /and the proposed change in corresponding author	Yunjian Li	04 13 2021
5th author	Cristina	Correia	I agree to the proposed new authorship shown in section 4 **/and the addition/removal*of my name to the authorship list** /and the proposed change in corresponding author	[illegible]	04122021
7th author	Zhiyang	Zhao	I agree to the proposed new authorship shown in section 4 **/and the addition/removal*of my name to the authorship list** /and the proposed change in corresponding author	Zhiyang Zhao	04 13 2021

SPRINGER NATURE

Change of authorship request form - Journals (pre-acceptance)

	First name	Family name		Signature	Date
8th author	Chengang	Zhao	I agree to the proposed new authorship shown in section 4 **/and the addition/removal*of my name to the authorship list** /and the proposed change in corresponding author	Chengang Zhao	04132021
9th author	Zhirong	Liu	I agree to the proposed new authorship shown in section 4 **/and the addition/removal*of my name to the authorship list** /and the proposed change in corresponding author	Zhirong Liu	04132021
10th author	Tao	Zhang	I agree to the proposed new authorship shown in section 4 **/and the addition/removal*of my name to the authorship list** /and the proposed change in corresponding author	Tao Zhang	04132021

Please use an additional sheet if there are more than 10 authors.

In case of author collaborations with formal agreement:

	Name of consortium/consortia	First name	Family name		Signature	Date
Representative/legal guarantor				I agree to the proposed new authorship shown in section 4 **/and the addition/removal*of my name to the authorship list** /and the proposed change in corresponding author		

Both added/removed authors should complete the information in the first table under Section 6.

---- End of form ----

SPRINGER NATURE Springer Nature is one of the world's leading global research, educational and professional publishers, created in May 2015 through the combination of Nature Publishing Group, Palgrave Macmillan, Macmillan Education and Springer Science+Business Media.

SPRINGER NATURE Change of authorship request form - Journals (pre-acceptance)

Section 6: Declaration of agreement. *All* authors, unchanged, new and removed *must* sign this declaration.
(NB: Please print the form, (docu)-sign and return/upload a scanned copy. Please note that signatures that have been inserted as an image file are acceptable as long as it is handwritten. Typed names in the signature box are unacceptable.) *** Please delete as appropriate. Delete all of the bold if you were on the original authorship list and are remaining as an author.**

	First name	Family name		Signature	Date
1st author	Zhiyou	Fang	I agree to the proposed new authorship shown in section 4 **/and the addition/removal*of my name to the authorship list** /and the proposed change in corresponding author	[signature]	0413 2021
2nd author	Hongzhi	Wang	I agree to the proposed new authorship shown in section 4 **/and the addition/removal*of my name to the authorship list** /and the proposed change in corresponding author	Wang Hongzhi	0413 2021
3rd author	Chao	Xu	I agree to the proposed new authorship shown in section 4 **/and the addition/removal*of my name to the authorship list** /and the proposed change in corresponding author	Chao Xu	0413 2021
4th authors	Saravut	Weroha	I agree to the proposed new authorship shown in section 4 **/and the addition/removal*of my name to the authorship list** /and the proposed change in corresponding author	[signature]	4/12/2021
5th author	Scott	Kaufmann	I agree to the proposed new authorship shown in section 4 **/and the addition/removal*of my name to the authorship list** /and the proposed change in corresponding author	[signature]	4/9/21
5th author	Haiming	Dai	I agree to the proposed new authorship shown in section 4 **/and the addition/removal*of my name to the authorship list** /and the proposed change in corresponding author	[signature]	4/9/21
7th author			I agree to the proposed new authorship shown in section 4 **/and the addition/removal*of my name to the authorship list** /and the proposed change in corresponding author		

SPRINGER NATURE Springer Nature is one of the world's leading global research, educational and professional publishers, created in May 2015 through the combination of Nature Publishing Group, Palgrave Macmillan, Macmillan Education and Springer Science+Business Media.
